# Human Cancer Cell Radiation Response Investigated through Topological Analysis of 2D Cell Networks

**DOI:** 10.1007/s10439-023-03215-z

**Published:** 2023-04-24

**Authors:** Luca Tirinato, Valentina Onesto, Daniel Garcia-Calderon, Francesca Pagliari, Maria-Francesca Spadea, Joao Seco, Francesco Gentile

**Affiliations:** 1 Department of Medical and Surgical Science, University Magna Grecia, 88100 Catanzaro, Italy; 2 Biological and Environmental Science and Engineering Division, King Abdullah University of Science and Technology (KAUST), Thuwal, 23955 Saudi Arabia; 3 Biomedical Physics in Radiation Oncology, DKFZ German Cancer Research Center, Heidelberg, Germany; 4 Department of Experimental and Clinical Medicine, Nanotechnology Research Center, University of Magna Graecia, 88100 Catanzaro, Italy; 5 Department of Physics and Astronomy, Heidelberg University, Heidelberg, Germany; 6 Institute of Biomedical Engineering, Karlsruhe Institute of Technology (KIT), Karlsruhe, Germany; 7 Department of Experimental and Clinical Medicine, University of Magna Graecia, 88100 Catanzaro, Italy

**Keywords:** Clonogenic assays, Radiation therapy, Radio-resistance, Cancer diagnosis, Topology, Small world networks, Networks analysis

## Abstract

Clonogenic assays are routinely used to evaluate the response of cancer cells to external radiation fields, assess their radioresistance and radiosensitivity, estimate the performance of radiotherapy. However, classic clonogenic tests focus on the number of colonies forming on a substrate upon exposure to ionizing radiation, and disregard other important characteristics of cells such their ability to generate structures with a certain shape. The radioresistance and radiosensitivity of cancer cells may depend less on the number of cells in a colony and more on the way cells interact to form complex networks. In this study, we have examined whether the topology of 2D cancer-cell graphs is influenced by ionizing radiation. We subjected different cancer cell lines, i.e. H4 epithelial neuroglioma cells, H460 lung cancer cells, PC3 bone metastasis of grade IV of prostate cancer and T24 urinary bladder cancer cells, cultured on planar surfaces, to increasing photon radiation levels up to 6 Gy. Fluorescence images of samples were then processed to determine the topological parameters of the cell-graphs developing over time. We found that the larger the dose, the less uniform the distribution of cells on the substrate—evidenced by high values of small-world coefficient (*cc*), high values of clustering coefficient (*cc*), and small values of characteristic path length (*cpl*). For all considered cell lines, $sw>1$ for doses higher or equal to 4 Gy, while the sensitivity to the dose varied for different cell lines: T24 cells seem more distinctly affected by the radiation, followed by the H4, H460 and PC3 cells. Results of the work reinforce the view that the characteristics of cancer cells and their response to radiotherapy can be determined by examining their collective behavior—encoded in a few topological parameters—as an alternative to classical clonogenic assays.

**Supplementary Information:**

The online version contains supplementary material available at 10.1007/s10439-023-03215-z.

## Introduction

Clonogenic assay—or colony formation assay—is the gold standard to assess the radioresistance (RR) and radiosensitivity (RS) of human cancer cells to external radiation fields and radiotherapy [5, 6, 12, 14, 16, 17]. This in vitro cell-survival-assay tests the ability of a single cell to grow into a colony. In this method, cancer cells are plated on a substrate, supplemented with cell culture medium, exposed to ionizing radiation, and monitored over time. Resulting values of plating efficiency (PE) and surviving fraction (SF) are then indicative of the ability of a cancer to withstand radiation. PE is the ratio between the final number of colonies and the initial number of cells. SF is the number of colonies expressed in terms of PE. Elevated values of radioresistance can impair the efficiency of radiotherapy—along with chemotherapy and surgery [24] one of the mostly used technique for cancer treatment [22, 23]. While classical clonogenic assays focus only on the number of colonies resulting from the treatment, the technique has evolved over time into higher levels of complexity. Recently, several groups [7, 8, 21, 31] have suggested to use the morphological characteristics of cells as a measure of RR or RS of cells. Inspired by this shift of paradigm, in a recent research paper [25] we have examined how the topological characteristics of lung cancer networks in 2D cultures correlate to ionizing radiation levels. Upon exposure to increasing radiation doses up to 8 *cpl*Gy, we found that the topological measures of H460, A549 and Calu-1 cell-graphs, including the characteristic path length (*cpl*) and the small world coefficient (*sw*), changed significantly. Higher values of *sw* than one were found in colonies treated with higher radiation doses and the larger the small world coefficient the lower the levels of radioresistance of the cells in the colony. These preliminary results point to the fact that the topological characteristics of 2D cultures are a clue of the nature of cancer cells and of their behavior in response to radiotherapy. Differently from conventional clonogenic assay where colonies are considered a *black box*, the method that we have developed examines in depth the characteristics of the systems trying to weave a thread between classical (colony number, cell number, cell density) and non-classical (clustering characteristics, small-world ness) cell-colonies parameters, and the properties of cancer cells. Nonetheless, while promising, results described so far are relative to a single cancer type, i.e. non-small cell lung carcinoma.

To demonstrate that the correlation between the small-world coefficient of cell graphs and the radiation dose is a common characteristic of cancer cells, we have here performed additional experiments using a variety of different cancer-cell lines, i.e. H4 epithelial neuroglioma cells, H460 lung cancer cells, PC3 bone metastatic cells of grade IV of prostate cancer and T24 urinary bladder cancer cells.

In this work, we put in culture cancer cells on conventional petri dish and measured the topological and morphological characteristics of the systems after exposure to increasing levels of radiation (0, 2, 4, 6 Gy). Cells were examined after 6 days from irradiation using conventional fluorescence microscopy, image analysis, and networks analysis techniques. Results of the analysis illustrate that the topology of all the considered cancer cell lines shows a very high sensitivity to the radiation dose—evidenced by values of small-world-ness that range in the 05–2 interval for the dose moving from 0 to 6 Gy. Data reported in the present work show that the topological and morphological traits of cell networks and radiation dose correlate for different types of cancer cells. Thus, the *topology* of cell-colonies can be a parameter used to describe the response of cells to radiation, evaluate the performance and of radiotherapy, determined the radio-sensitivity and radio-resistance of cancer cells.

## Methods

### Cell Cultures

Human neuroglioma (H4), prostate adenocarcinoma (PC3), urinary bladder transitional carcinoma (T24) and lung carcinoma (H460) cells were purchased from ATCC. H460 were cultured in RPMI 1640 medium (Thermo Fischer Scientific), T24 in McCoy’s 5a medium (Thermo Fischer Scientific), PC3 in F-12K medium (Thermo Fischer Scientific), and H4 in DMEM medium (Thermo Fisher Scientific). Cell doubling times for each cell line were calculated in sub-confluent conditions (data not shown). The results were: 21 h for the H460, 29 h for the T24, 31 h for PC3, and 22 h for the H4 cell lines. All media were supplemented with 10% Fetal Bovine Serum (Thermo Fischer Scientific) and 1% Pen/Strep (Thermo Fischer Scientific) and cells were maintained at 37 °C in a humidified 5% CO_2_ atmosphere. All cell lines were authenticated by means of Multiplex human Cell line Authentication (MCA) assay and tested for mycoplasma contamination by EZ-PCR Mycoplasma Test Kit (Biological Industries).

### Clonogenic Assay and Staining

A clonogenic assay was run for all cell lines to investigate their clonogenic potential as reported elsewhere [25]. Briefly, H460, T24, PC3, and H4 were seeded in six-well plates at a density of 300, 600, and 3000 cells/well after being irradiated with 2, 4, and 6 Gyy X-rays, respectively, with a Multi Rad 225 kV irradiator (Faxitron Biotics) together with control (untreated) cells (200). Soon after the radiation treatment, cells were kept for 6–8 days in a 37 °C incubator with a 5% CO_2_ humidified atmosphere. The different parameters like cell doubling times, the number of seeded cells, and radiation doses were all considered for the experiment output. In fact, all clonogenic assays were stopped after 6–8 days depending on the cell line to obtain colonies with a similar number of cells and avoid cell overgrowth or an excessively cell lost. At the end of the incubation times, all samples were fixed in 100% ethanol for 4 min and stained using a $1 \mathrm{\mu g}/\mathrm{mL}$ Hoechst 33,342 (Thermo fisher Scientific). The plates were stored at 4 °C until imaged.

### Fluorescence Imaging

All samples were imaged with an inverted fluorescence microscope (Nikon Eclipse Ti2-E) using a Nikon Plan Fluor 10X or a Nikon S Plan Fluor ELWD 40× objective. The 14-bit Images obtained, with a pixel resolution of 0.7373 × 0.7373 µm^2^ (10X) or 0.1834 × 0.1834 µm^2^ (40X), were recorded with a Nikon Ds-Qi2 Camera.

### Fluorescence Image Segmentation

More than 2500 images of H4, H460, PC3 and T24 cells treated at 0, 2, 4, 6 Gy were analyzed with Matlab® (2017b) to extract cell shape descriptors and network parameters following the methods reported in reference [25] (Scientific Reports 2022). Before the topological analysis, images were converted to grayscale, low-pass filtered, and then binarized with Otsu’s method [20]. After black and white conversion, images were segmented by a watershed transformation [15] followed by distance transform [13]. Details of the segmentation process are reported in the Supporting Information 1.

### Generating Cancer-Cell Graphs

Upon cell-image segmentation, cell centers were connected using the well-established Waxman model [30]. The Waxman algorithm determines whether cell nodes are linked on the ground of an inverse-distance rule: the smaller the distance ($\delta$) between cell-centers, the higher the likehood *P* that cells are connected, where $p=\alpha { e}^{-\delta /\beta l}$, $l$ is a reference length, i.e. the maximum inter-cellular distance in the image, and $\alpha =1$ and $\beta =0.025$ are model parameters. After comparing *P* to a threshold value *P*, the algorithm establishes whether cells of the image are connected ($1-p<P$) or not ($1-p>P$) (Supporting Information 2 and reference [25]). In this study *P* is a constant that we have chosen being 0.9, 0.95 and 0.98.

### Topological Analysis of Cell Networks

Cancer cell graphs generated by the Waxman algorithm were analyzed using the methods of networks-science described in the Supporting Information 3. For each of the considered cancer-cell networks, we determined the graph-degree (*k*), the clustering coefficient (*cc*), the characteristic path length (*cpl*), and the small world coefficient (*sw*). The significance of these parameters is recapitulated in the Supporting Information and recalled throughout the paper: they provide a complete description of the network topology. While in this study we have examined cell-graphs using the complete set of variables *k*, *cc*, *cpl* and $\mathrm{SW}$, however for the discussion and conclusions we did focus on the sole SW variable. The small-world (SW) coefficient offers some advantages over other cited metrics. Firstly, it is a non-dimensional parameter. Since SW is determined by comparing a real network G to an equivalent random network with the same size of G, it shows a very low sensitivity to the number of cells laying on a substrate. Thus, it represents a robust estimate of the topology of a system regardless of its size or scale. In the second place, SW is determined as the combination of the clustering coefficient (*cc*) and the characteristic path length (*cpl*). Thus, it incorporates 2 different metrics and exhibits the predictive power of both *cc* and *cpl*. Lastly, the small-world coefficient is a very well-studied metrics used to characterize real networks, such as biological, industrial, social networks. As a result, there exists a great body of comparative literature, case studies and reports.

### Statistical Analysis

Results of the study are reported as mean ± standard deviation. The parameters of the cell-graphs including the graph-degree, the clustering coefficient, the characteristic path length, and the small world-ness were determined from over 50 fluorescence images per cell-type and dose. Comparison between sample means was performed using a Student’s *t*-test statistics (two-tailed, unpaired), where the null hypothesis of the equivalence of sample means was verified under the assumption that elements in each group are normally distributed. *P*-values resulting from the Student’s *t*-test below than 0.05 are indicative of a statistically significant difference between sample groups.

## Results

### Acquiring Images of Irradiated Cancer-Cell Lines and Image Analysis

**Fig. 1:**
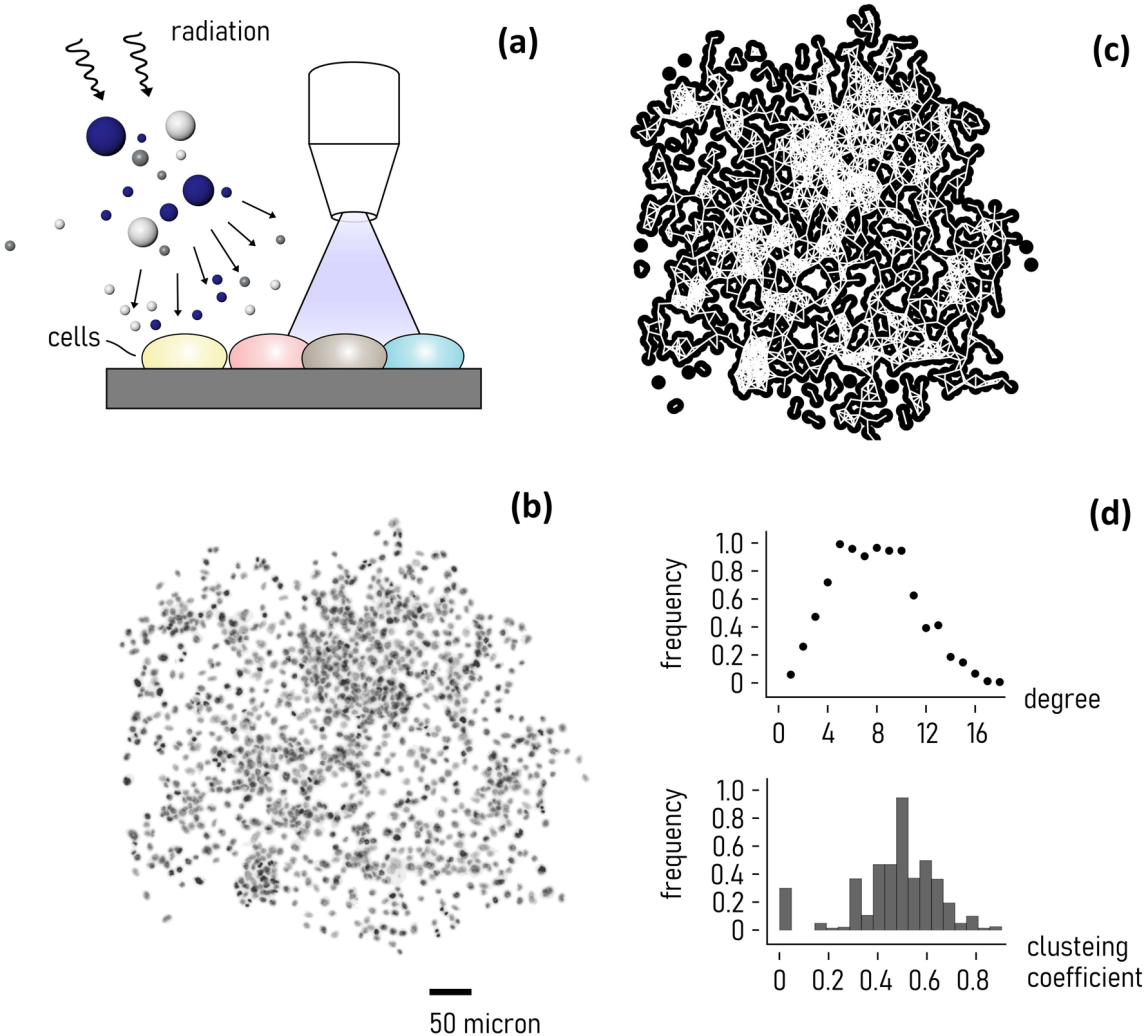
*Schematics of the experiments*. Cancer cells (H4, H460, PC3, T24) were plated on a petri dish, subjected to different levels of radiation, i.e. 0, 2, 4, 6 Gy, and left in incubation for 6 to 8 days. After staining, cells and the cell-layout were imaged using fluorescence microscopy (**a**). Resulting fluorescence images were processed using standard image-analysis algorithms to locate in each image the cells and the cell-boundaries (**b**). Cell networks generation: cell centers were connected using the Waxman algorithm—a cell-graph was associated to each cell layout (**c**). Properties of resulting networks were determined using the methods of networks science, in the example reported in (**d**) we report the degree and clustering coefficient distribution relative to the network shown in (**c**)

 H4 epithelial neuroglioma cells, H460 lung cancer cells, PC3 bone metastatic cells of grade IV of prostate cancer and T24 urinary bladder cancer cells were seeded into 6-multiwell plates at an initial density of 200, 300, 600, 3000 cells/well relative to the 0, 2, 4 and 6 Gy X-ray irradiation exposure, respectively. Irradiated cells were kept in culture for 6 days, and then fixed in ethanol, stained with Hoechst 33342, and imaged using standard fluorescence microscopy (Fig. 1a). Resulting fluorescence images (Fig. 1b) were then processed using standard image analysis algorithms described elsewhere [25] (methods), converted into black & white, watershed transformed and segmented. Individual cells in each image were then linked using the Waxman algorithm [25, 30] (Fig. 1c). The algorithm connects pairs of cells with a probability *P* that scales with the inverse of their distance, and by comparing *P* to a threshold value *P*, such that if $p-P>1$, cells are connected. This enabled to associate to each colony of cells a graph, or network. Cell-graphs were then conveniently analyzed using networks science to extract the most salient topological features of the networks (Fig. 1d), including the local and global node degree (*k*), the local and global clustering coefficient (*cc*), the characteristic path length (*cpl*), and the small world coefficient (*sw*). These parameters are frequently used to describe network topology, capturing most of their characteristics [2, 18, 19, 28, 29]. The definition and significance of *k*, *cc*, *cpl* and *sw* are reviewed in a separate supporting information file. Here it is useful to recall that the clustering coefficient is a measure of local and global connectivity, it is the proportion of existing links in the proximity of a node, to the total number of links that can be possibly secured around that node. The characteristic path length is the average distance between nodes of a network. According to the categorical definition of Humphries [9], the small-world coefficient is a *combination* of *cc* and *cpl*. It measures how a real network *g* compares to a random network *R* of the same size of *g*. If $sw>1$, elements of *g* are more clustered and the paths shorter than in *R*. This also indicates that signals and information can be possibly transferred more efficiently in *g* than in *R* [1, 3, 11, 18, 19].

### Determining the Number of Cells Post Irradiation

**Fig. 2:**
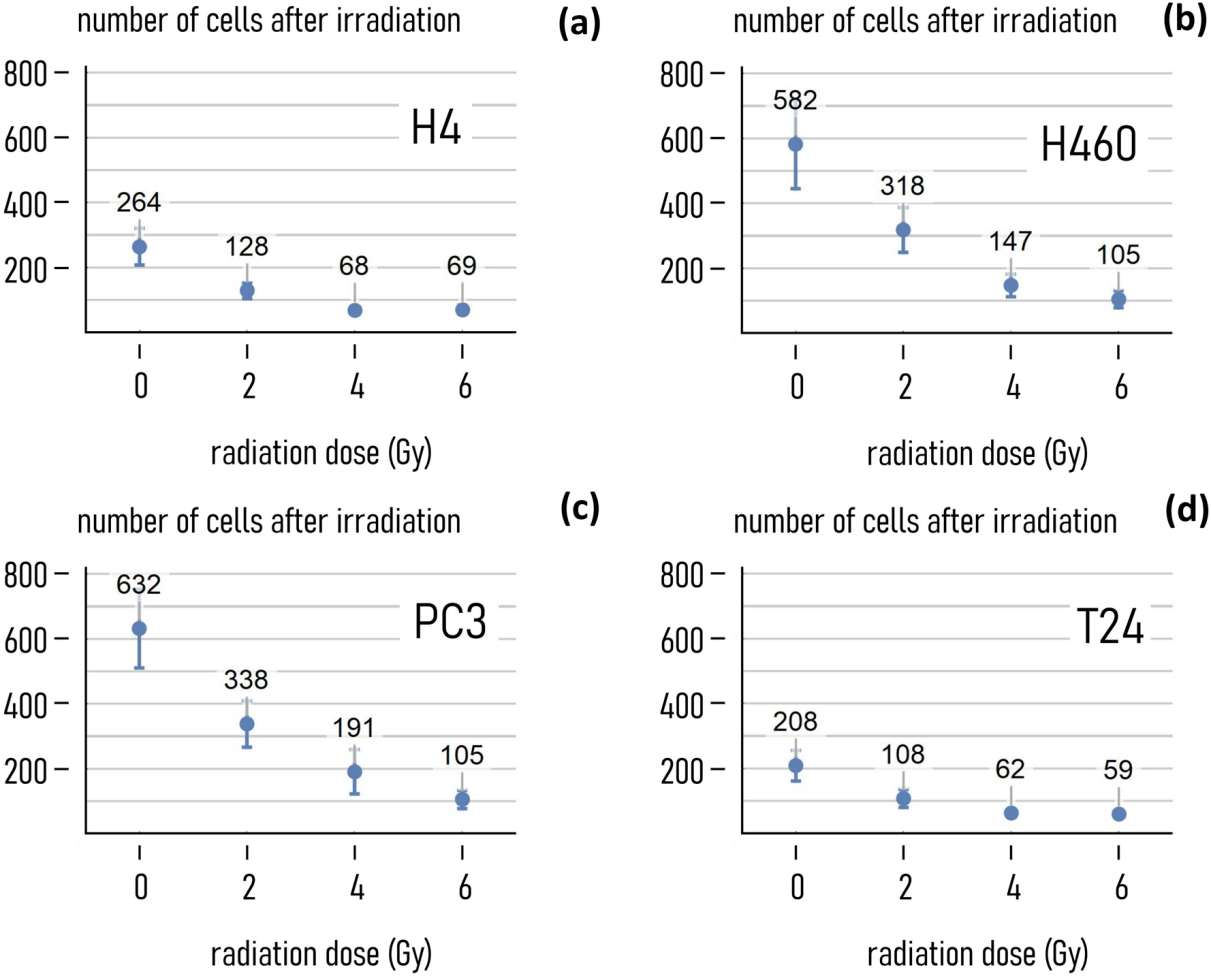
*Cell number upon exposition to radiations*. Average number of cells measured on a region of interest upon exposure to different levels of radiation (0, 2, 4, 6 Gy) for different cancer-cell lines, i.e. H4, H460, PC3, T24

Post-processed cell images were, in the first place, analyzed to determine the average number *n* of cells in the field of view as a function of the radiation dose *d*. *n* is higher for non-treated cells ($d=0 Gy$), with values of *n* ranging from 208 for the T24 cell line to 630 for the PC4 cells (Fig. 2). For all the considered cell lines, *n* then smoothly decreases with *d*. This reduction is significant in the $0-4 \mathrm{Gy}$ range, and is less significant in the $4-6 \mathrm{Gy}$ range. Consider the H4 cells: for these, the number of cells in the field of view varies from 262 to 128, and from 128 to 68 for *d* moving from 0 to 2 Gy, and from 2 to $4 \mathrm{Gy},\mathrm{ respectively}$. Thus, the value of *n* halves every 2 Gy in the 0–4 Gy interval. In contrast, moving from 4 to 6 Gy, *n* transitions from 68 to 69—i.e. with no appreciable change. This behavior is common to all considered cell lines. In the $0-4 \mathrm{Gy}$ interval: the number of imaged cells experiences a decrease of $\sim 74\%$, $\sim 74\%$, $\sim 69\mathrm{\%}$ and $\sim 70\%$, for the H4, H460, PC3, T24 cell lines, respectively. In the 4–6 Gy interval:* n* experiences a decrease of ~ 0%, ~ 28%, ~ 45 and ~ 4% for the same cell lines. In any case, the number of cells in the region of interest is higher than 59.

### Topological Analysis of Samples

**Fig. 3:**
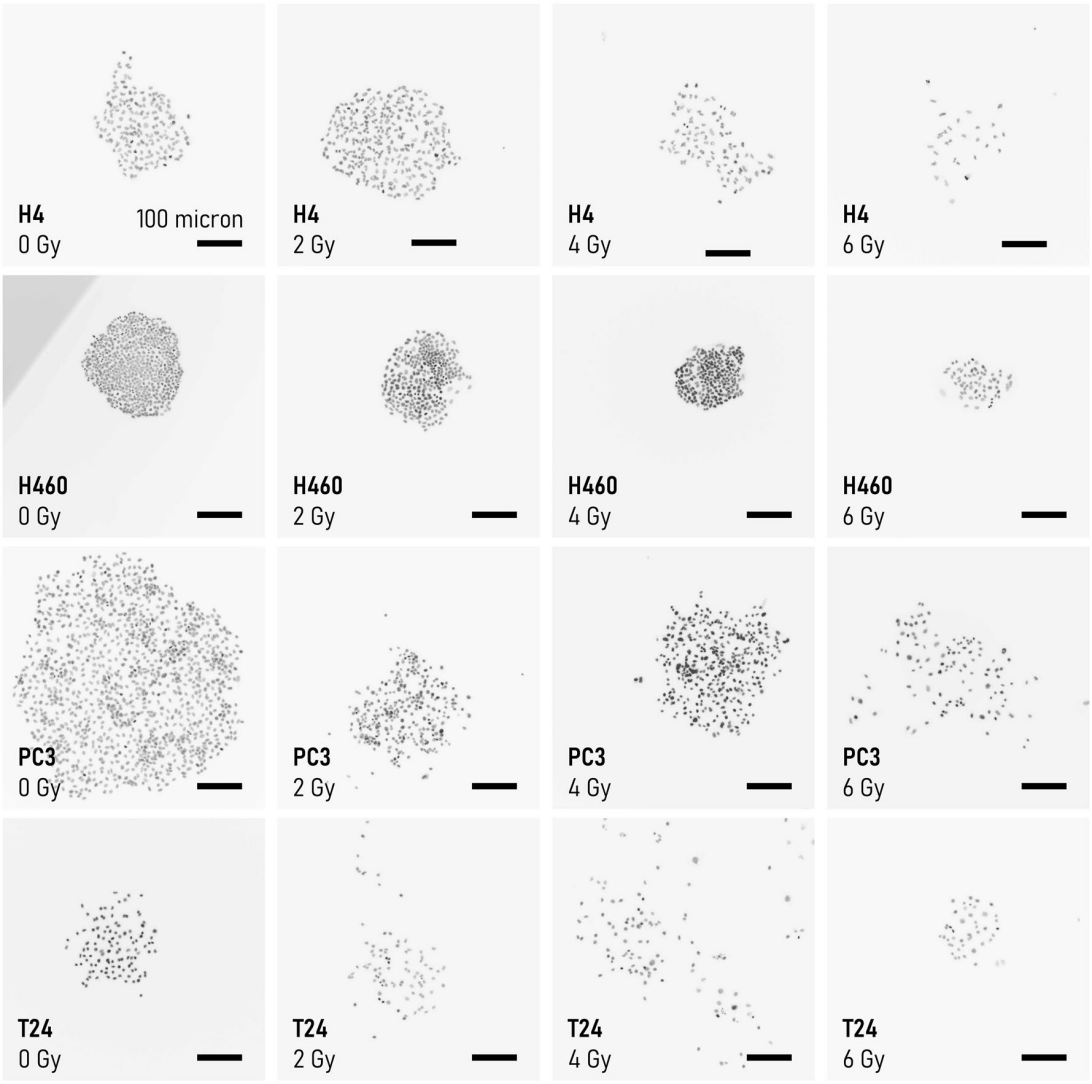
*Effect of radiations on the cell-layout*. Examples of cell-layout after exposition to different levels of radiation, 0, 2, 4, 6 Gy, for different cancer-cell lines. For a fixed cell line, the larger the levels of radiation, less continuous and uniform the distribution of cells on the substrate

**Fig. 4:**
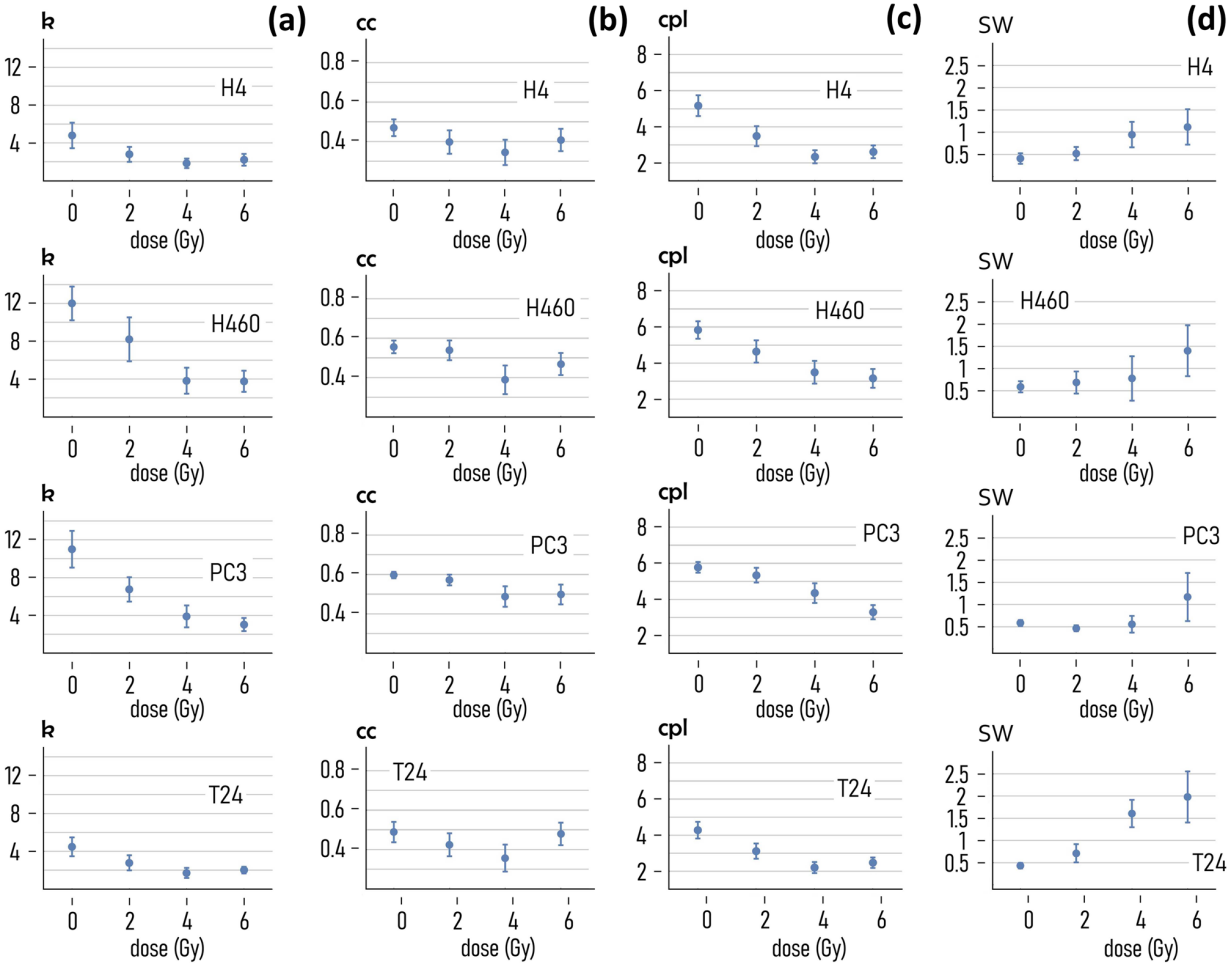
*Effect of exposition to radiation on the topology of cancer-cell graphs*. Mean values of the degree of a graph *k* (**a**), the mean clustering coefficient *cc* (**b**), the characteristic path length *cpl* (**c**), and of the small-world coefficient *sw* (**d**) measured in cell-networks resulting from exposition to different radiation levels (0, 2, 4, 6 Gy) for different cancer-cell lines (H4, H460, PC3, T24). (The parameters used in the Waxman model to wire the cell-graphs are $\alpha =1$, $\beta =0.025$, $P=0.95$)

In Fig. 3 we report representative images of the cell appearance for each considered cell line after exposure to specific levels of ionizing radiation. One can observe that at lower values of radiation, the distribution of cells in a colony is uniform. At higher degrees of radiation, the disposition of cells in a colony is less regular, and the cell-density varies in the field view. This suggests that the topological properties of 2D systems of cells are not preserved under exposition to external radiation fields, and that a topological measure of cell-graphs can be indicative of the nature of cancer cells and of their ability to resist to radiotherapy. We used networks science to determine quantitatively the topological characteristics of graphs generated from the fluorescence cell images (methods). Examples of graphs generated from cell images for all considered cell-lines and exposing dose are reported in a separate Supporting Information 4. For each cell line and radiation dose, we examined more than 40 cell-graphs (technical repeats), from which we estimated the degree of a graph (*k*), the clustering coefficient (*cc*), the characteristic path length (*cpl*), the small world coefficient (*sw*). The average values of *k*, *cc*, *cpl* and *sw* are reported in the diagrams in Fig. 4 for each combination of cell line and dose. We determined the values of the parameters using a probability $P=0.95$ as a threshold in the Waxman (network-generator) algorithm. Data reported in Fig. 4a indicate that the degree of the cell-network decreases with the dose for all the considered cell lines. The degree of a graph is the average number of links per node. A similar decrease is more relevant for H460 and PC3, with *k* moving from ~ 12 to ~ 4, and from ~ 11 to ~ 3 respectively, in the $0-6 Gy$ interval. It is less relevant for H4 and T24 cells, for which *k* transitions from ~ 5 to ~ 2 (H4 cells), and from ~ 4 to ~ 2 (T24 cells), in the considered radiation-dose interval. Thus, larger radiation levels impair the ability of cells to establish connections. The clustering coefficient of cell-graphs is reported in Fig. 4b. Differently from *k*, the clustering coefficient is more stable in the $0-6\mathrm{ Gy}$ interval. Values of *cc* oscillate between $cc\sim 0.35$ and $cc\sim 0.5$ for the H4 cells, $cc\sim 0.4$ and $cc\sim 0.55$, for the H460 cells, between $cc\sim 0.5$ and $cc\sim 0.6$ for the PC3 line, $cc\sim 0.35$ and $cc\sim 0.5$ for the T24 cells. Moreover, values of the clustering coefficient correlate only moderately with the dose: for all the considered cell lines, *cc* decreases linearly with* d* in the $0-4 \mathrm{Gy}$ interval while after the 4 Gy limit values of *cc* rise again (H4, H460, T24 cell lines) or plateau (PC3 line). In contrast to the clustering coefficient, the characteristic path length shows a very high sensitivity to the dose (Fig. 4c). For H4, the v variable changes from an average value of $cpl\sim 5$ steps, evaluated at $0\mathrm{ Gy}$, to $cpl\sim 2.5$ determined at $6 \mathrm{Gy}$. A similar trend is observed for the remaining cell lines: the mean values of *cpl* vary between $cpl\sim 6$ and $cpl\sim 3$, for H460 and PC3 cells, and between $cpl\sim 4$ and $cpl\sim 2$ for T24 cells. Thus, much of the variation of topology of cell-networks—as a function of the dose—is ascribable to the distance between the network elements. The combined effects of *cc* and *cpl* are incorporated by the small world coefficient (*sw*). Values of the small world coefficient different from one indicate that the network topology departs from that of uniform-random Erdos–Renyi models. The *sw* for the considered cell lines and radiation doses is reported in Fig. 4d. Data indicate that the small-world coefficient increases with the dose. For H4, H460 and PC3 cells, *sw* is higher than one for doses greater than or equal to $4 \mathrm{Gy}$. For T24, *sw* is higher than one for doses greater than $2 \mathrm{Gy}$.

**Fig. 5:**
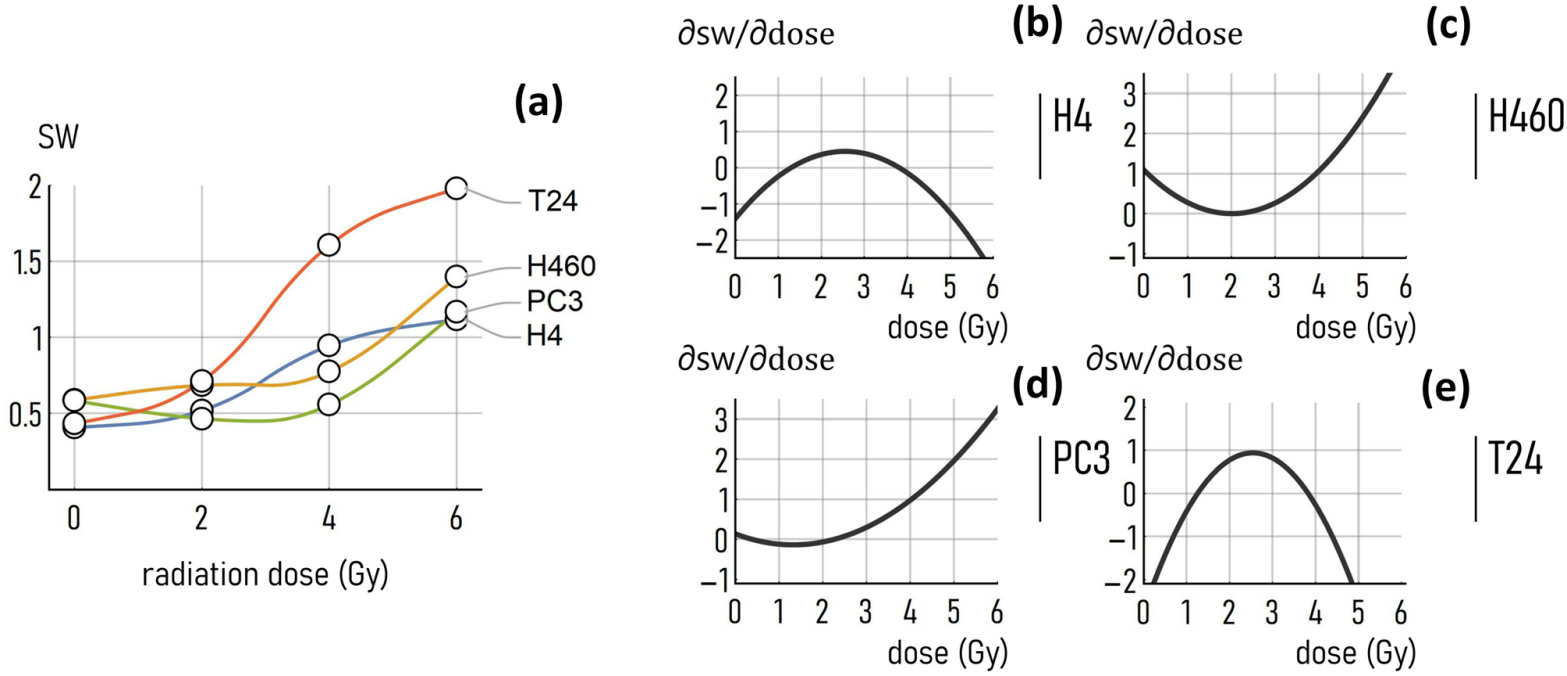
*Comparing the small-world-ness among different cell lines*. Value of the small-world coefficient determined as a function of the dose (0, 2, 4, 6 Gy) for all considered cell lines (H4, H460, PC3, T24) (**a**). Sensitivity of the small-world-coefficient against the dose, $s=\partial sw/\partial dose$, calculated for the H4 (**b**), H460 (**c**), PC3 (**d**), T24 (**e**) cell-lines

To highlight even further differences or similitudes between cancer cell lines, we have reported in the same diagram the *sw* coefficient determined for all cells considered in this study. Moreover, we have determined the sensitivity of *sw* to the dose, calculated as $s=\partial sw/\partial \mathrm{dose}$, for all considered cell-lines and in the $0-6 \mathrm{Gy}$ (Fig. 5). Results indicate that, while the larger values of small-world-ness are associate to the T24 cancer cell line (Fig. 5a), the H460 and PC3 cells exhibit the highest sensitivity to the dose, with values of sensitivity as high as $s=3 sw \mathrm{units}/\mathrm{Gy}$ (Fig. 5c, d). Notably, the values of sensitivity depend, in turn, on the values of radiation dose for which they are determined. While the H4 and T24 cells show a higher sensitivity on the dose in the 0–4 Gy interval (Fig. 5b, e), in contrast, for the H460 and PC3 cells the larger the value of the dose the more relevant the effect of external radiative fields (Fig. 5c, d). Notably, diagrams as those reported in Fig. 5b–e may provide a clear indication to the radiation therapist on how to regulate the intensity of the dose. More sophisticated evolutions of these design maps that will be developed over time, can represent a guidance for the treatment and characterization of cancer cells—based on the topological analysis of systems of cells.

**Fig. 6:**
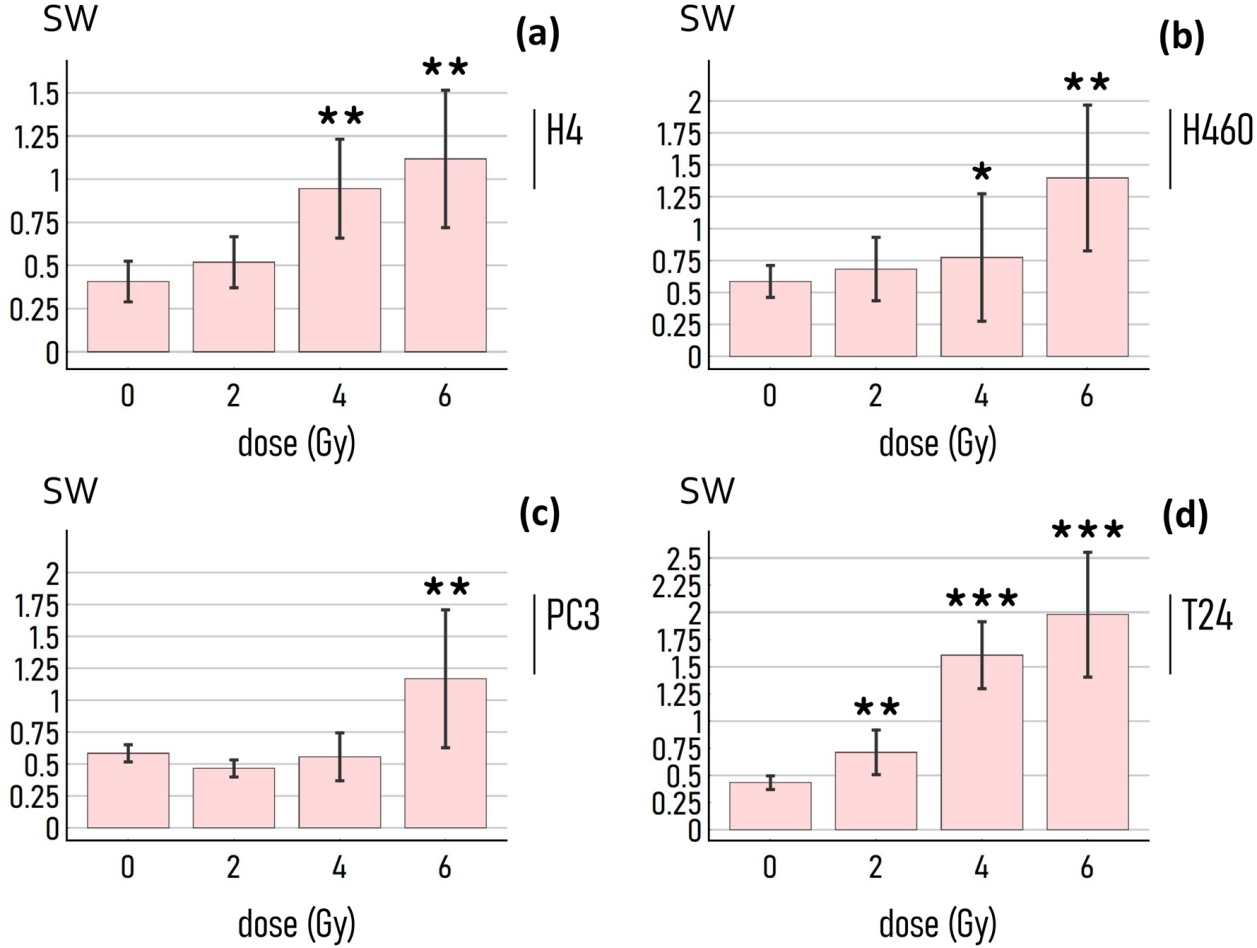
*Statistical significance test*. We used a Student’s test to examine whether the small-world-ness measured in cancer-cell graphs correlates to the values of exposing radiation. The *sw* coefficient of cell-networks deviates significantly from the control after exposure to radiation levels higher or equal to 4 Gy for the H4 (**a**), H460 (**b**), T24 cells (**d**), and to doses higher or equal to 6 Gy for the PC3 cells (**c**). In the diagrams, the stars flag levels of significance of the statistical test: if the p-value is less than 0.05, it is flagged with one star (*); if the p-value is less than 0.01, it is flagged with 2 stars (**); if the p-value is less than 0.001, it is flagged with 3 stars (***). (The parameters used in the Waxman model to wire the cell-graphs are $\alpha =1$, $\beta =0.025$, $P=0.95$)

We performed a Student's t test between sample means to examine whether the small-world characteristics of cell-graphs are statistically affected by the external dose (Fig. 6). Results of the analysis indicate that the difference between the small-world-coefficient of networks of H4 cells subjected and not subjected to a radiation of 4 Gy and 6 Gy is statistically significant with a calculated $P \mathrm{value}<0.05$. In the same way we determined that the radiation levels for which the small-world-coefficient of H460 cells is statistically significantly different from the control are 4 Gy with a $P \mathrm{value}<0.05$ and 6 Gy with a $P \mathrm{value}<0.01$. The critical dose for the PC3 cells was determined as $d=6 \mathrm{Gy}$ ($P \mathrm{value}<0.01$). For the T24 cells, the critical doses correspond to 2 Gy ($P \mathrm{value}<0.01$), 4 Gy ($P \mathrm{value}<0.001$) and 6 Gy ($P \mathrm{value}<0.001$) (a full comparative statistical analysis between samples is reported in a separate Supporting Information 5—including values of sample size, *P*-value,* t*-stat, and degree of freedom, for all considered cell lines and exposing dose).

The values of small-world-ness that we have found are relative to networks generated using a probability threshold $P=0.95$ in the Waxman model. We performed additional analysis to demonstrate that the $sw-dose$ relationship that we have determined for $P=0.95$ and illustrated in Figs. 4, 5 and 6 is a common characteristic of cancer cells and is only moderately affected by *P* and the network generator rule. Diagrams in the Supporting Information Fig. 6.1 illustrate the small-world-ness *sw* of cell-graphs built on different values of *P*, i.e. $P=0.9$, $P=0.95$, $P=0.98$, for the H4, H460, PC3, T24 cell lines, as a function of the radiation dose. For all the considered wiring threshold probabilities *P* the values of *sw* steadily increase with the dose—and the patterns of variation of *sw* are similar. Diagrams in the Supporting Information Fig. 6.2 show how the small-world coefficient of cancer cell graphs varies as a function of the cut-off probability *P*—for different values of the radiation dose.

Diagrams illustrate that, for smaller values of the dose comprised between 0 and 2 Gy, the *sw* coefficient increases moderately with *P*. In contrast, for larger values of the dose (4, 6 Gy) the *sw* coefficient decreases moderately with *P*, with the exception, notably, of the H460 cells—for which *sw* always increases with the probability *P* regardless of the dose. This is easily explained considering that—for a sufficiently high number of cells (thus smaller dose)—heightening the cut-off probability has, as a consequence, a reduced number of links in the network leading to sparse clustered networks, reflected by large values of *sw*. When the number of cells is excessively low (thus higher dose), increasing the cut-off probability has as an effect the creation of isolated clusters in the networks, disconnected from the others, and thus a reduced value of *sw*.

Collectively, data reported in the Supporting Information Fig. 6 suggest that—while the topology of networks of cells is sensitive to the dose and the nature of the cells—it is barely influenced by the parameter of the network generator model.

### Morphological Analysis of Samples

**Fig. 7:**
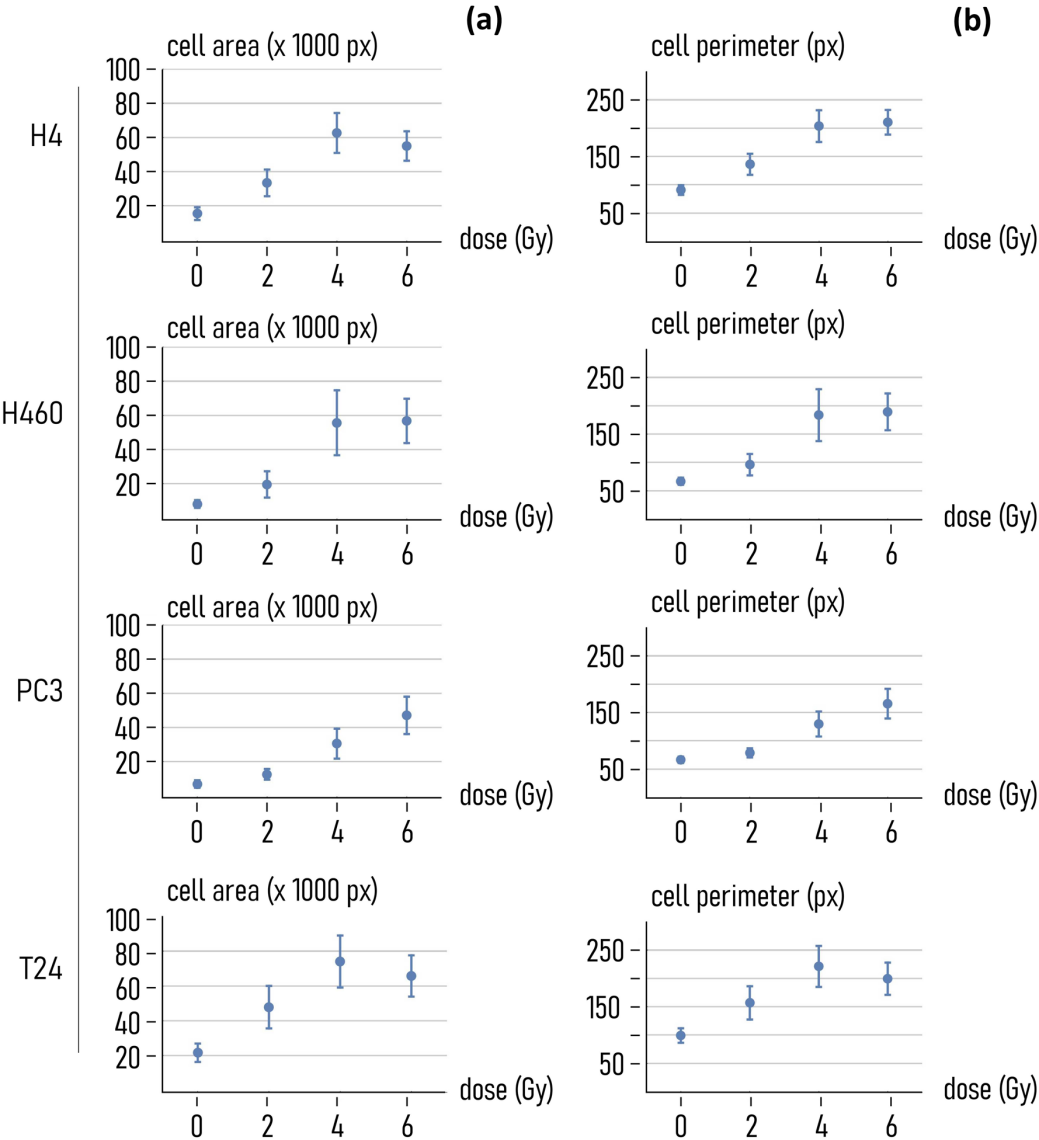
*Morphological analysis of samples*. Mean cell area (**a**) and cell perimeter (**b**) as a function of the radiation dose, calculated for different cell lines (H4, H460, PC3, T24)

The sensitivity of cancer-cells to the dose is also confirmed by morphological analysis of samples. Using the methods reported in a separate supporting information, we have determined for each cell-line and exposing dose the average value of the area (Fig. 7a) and perimeter (Fig. 7b) of the cells in the field of view. The area is expressed in pixel units, the perimeter in pixel edges. For all cell lines, the cell area increases linearly in the 0–4 Gy range and attains to a steady state value or decreases between 4 and 6 Gy, with the exception of the PC3 cells, for which the area continues to grow also in the 4–6 Gy interval. Notably, in the 0–6 Gy range the area of the cells increases of ~ 2.5 (H4 cells), ~ 6 (H460 cells), ~ 6 (PC3 cells), 2 (T24 cells) times, depending on the cell-line. The perimeter of the cells follows a similar trend: in the 0–6 Gy interval the perimeter of the cells experiences upward variations of ~ 130% (H4 cells), ~ 180% (H460 cells), ~ 150% (PC3 cells), 100% (T24 cells).

These results deserve to be discussed even further. While Fig. 3 suggests—at a glance—that the area of cells in the field of view is decreasing, in fact it indicates that the number of cells diminishes with the dose, as correctly illustrated in Fig. 2. The measure that we report in Fig. 7 of the paper is the area of individual cells—determined by post processing and analysis of cell images as described in the method. Therefore, data reported in Figs. 2, 3 and 7 are not contradictory and illustrate that, for increasing radiation dose, while the number of cells on the sample surface decreases the area of individual cells on the same surface increases. These opposite, seemingly contradictory trends both indicate that high radiation levels impact on cell internal machinery.

## Discussion

### Surpassing the Limits of Classical Clonogenic Assays

The existing clonogenic protocols [6] aim to determine the RS and RR of cells by examining the number of cell-colonies forming on a bidimensional support upon irradiation. This number is connected to the properties of cells, such as the cell duplication time and senescence, cell motility, the cell-surface and cell–cell adhesive characteristics. Since radiotherapy damages the DNA of cells, with a consequent deterioration of cellular functions, by measuring the number of colonies in a sample one can estimate the extent of DNA damage and the effects of radiation on cells. However, there are at least two problems with this experimental approach.

The first is that it is predominantly phenomenological. Since the concerted effects of the described cellular functions and radiation on cells are not completely understood, any model that puts in relationship the number of colonies to the cell radiosensitivity can only be empirical, based on observation and experiment. This also implies that any related data to an experimental campaign aimed at characterizing the radiotherapy properties of a cell-line are limited and conditional to the experimental conditions, and offer little perspective. The second, is that the number of colonies in a sample is a derived unit. It is a very averaged and coarse measure of how cells behave in response to radiotherapy: it encapsulates an otherwise complex phenomenon—with possible loss of information.

The method that we have devised is an attempt to surpass the limitations found in conventional clonogenic assays. It uses topology to investigate the nature of cancer cells subjected to radiation. The topology of a great many of cells in a colony is determined by the knowledge of the position of cell-centers, thus it represents a more intimate, more complete, and less qualitative description of a system, compared to a mere count of cells or cell-colonies. The cancer cell-graphs that we have analyzed in this study result from the interaction of all the elements in a colony, as such they convey the maximum information about a system of cells, and the cell-networks variables (*k*, *cc*, *cpl*, *sw*) are a robust and reliable description of how external factors, i.e. radiation, impact on that system. Robustness of the method is evidenced by the very low sensitivity of the small-world-coefficient of graphs to *P*, that is a parameter in the Waxman cell-graph generator (Supporting Information 6). Reliability of the method is proven by a consistent trend of the $sw-dose$ curve, for different cancer cells: for all considered lines (H4, H460, PC3, T24) the small-world-coefficient increases with the dose, the variability of the rate of change may reflect a variability of radioresistance or radiosensitivity among cell lines (Fig. 5). Notably, the method described in this paper examines the cells and connections within a colony and disregards the number of colonies in a plate. This shift of paradigm and scale may possibly improve the practice and increase the efficiency of clonogenic assays, and the measure of the effects and consequences of radiotherapy on cancer cells.

### Topological and Morphological Analysis Results are Consistent

A vast body of existing literature has described the effect of external radiation fields on cell morphology. Notably, results of these reports are not contradictory, and all illustrate that high radiation levels have, as a consequence, an increase of the area of cells adhering on the sample surface [10, 21, 26, 27], possibly due to the biological adaptation of tumor cells to radiation therapy [4]. The morphological analysis of cells that we have made in our study is not—thus—innovative per se, and is more a confirmation of the correctness of the experimental methods and procedures.

While it is very well understood that ionizing radiation influences cell-morphology, the network-analysis that we have carried out in this work demonstrates that radiations have *also* a measurable effect on the topology of cell colonies. This study represents one of the first empirical attempts to join seemingly uncorrelated variables, such as the topology of colonies, the number of cells in colony and cell morphology. For the cell-lines considered in this study, the area (morphology) and the small-world characteristics (topology) of cancer-cells of individual cells increase with the dose, while the number of colonies and number of cells in a colony (clonogenic assay) decrease with the radiation levels. The specific way in which variables are correlated is described in the diagrams of the work and depends on the cancer-cell line under analysis.

While the reason why radiation causes cell changes at the micro (morphology) and macro (topology) scale may depend on complex biological mechanisms and has to be examined in specific studies—we can speculate that any cause affecting the elements of a system (cells) has inevitable consequences on the system itself (cell-network). High radiation levels hamper adhesion and proliferation of cells on a substrate, evidenced by larger values foot-area and smaller values cell density, compared to un-treated cells. A reduced number of cells has, as a consequence, a more discontinuous/less-uniform distribution/layout of elements on the sample surface. Resulting cell-graphs are thus significantly diverse from uniform random graphs, as evidenced by higher values of small-world coefficient and statistically different values of other topological measures, such as the clustering coefficient and the characteristic path length.

## Conclusions

In this study, we have examined whether external radiation fields have an effect on the topology of H4, H460, PC3 and T24 cells, i.e. different cancer types. After exposing cells to radiations with intensity as high as 6 Gy, we measured the topological characteristics of resulting cell-graphs using fluorescence microscopy, standard image analysis algorithms, and networks analysis. Results illustrate that the larger the dose, the higher the values of small-world coefficient (*sw*) and of clustering coefficient (*cc*), and the smaller the characteristic path length (*cpl*). For all considered cell lines, $sw>1$ for doses higher or equal to 4 Gy. Results of the work suggest that the internal topology of a colony of cells can be a clue of the radiosensitivity and radioresistance of cells. This concept can be possibly translated to clinical practice. Portions of tissue taken within a tumor through solid biopsy can be dissociated—and resulting cancer-cells plated on a support. Cell-colonies developing over time will be subjected to increasingly high radiation levels and examined using the methods described in this study. The values of small world determined for such samples will be then compared to the values found for non-cancer cells of the same tissue/organ. From the comparison, one will dissect whether cells from the patient are radiosensitive or radio-resistant, and to which extent—key determinants in tailoring the right treatment for the patient.

## Supplementary Information

Below is the link to the electronic supplementary material.

Supplementary file1 (PDF 3470 kb)

## Data Availability

Research data and related metadata are available upon reasonable request to the corresponding author.
