# Supplementary material for: Human Cancer Cell Radiation Response Investigated through Topological Analysis of 2D Cell Networks
Source: Ann Biomed Eng. 2023 Apr 24;51(8):1859–71. doi: 10.1007/s10439-023-03215-z (PMC10326123; doi:10.1007/s10439-023-03215-z)
Supplement: Supplementary file 1 — Supplementary file1 (PDF 3470 kb) [file 10439_2023_3215_MOESM1_ESM.pdf]

Human Cancer Cell Radiation Response Investigated through Topological Analysis of 2D Cell-Networks

Luca Tirinato [1, 2, 3] *, Valentina Onesto [4,] *, Daniel Garcia-Calderon [3, 5], Francesca Pagliari [3], Maria-Francesca Spadea [6, 7], Joao Seco [3, 5] †, Francesco Gentile [4, 7] †

[1] Department of Medical and Surgical Science, University Magna Grecia, 88100 Catanzaro, Italy

[2] Biological and Environmental Science and Engineering Division, King Abdullah University of Science and Technology (KAUST), Thuwal 23955, Saudi Arabia

[3] Biomedical Physics in Radiation Oncology, DKFZ German Cancer Research Center, Heidelberg, Germany

[4] Nanotechnology Research Center, Department of Experimental and Clinical Medicine, University of Magna Graecia, Catanzaro 88100, Italy

[5] Department of Physics and Astronomy, Heidelberg University, Heidelberg, Germany

[6] Institute of Biomedical Engineering, Karlsruhe Institute of Technology (KIT), Karlsruhe, Germany

[7] Department of Experimental and Clinical Medicine, University of Magna Graecia, Catanzaro, 88100, Italy

* These authors contributed equally to this work

† Corresponding Authors: j.seco@dkfz-heidelberg.de, francesco.gentile@unicz.it

Supporting Information

**Supporting Information 1**	*Image segmentation and analysis*	Pg. 2
**Supporting Information 2**	*Generating networks using the Waxman algorithm*	Pg. 3
**Supporting Information 3**	*Topological analysis of cell networks*	Pg. 4
**Supporting Information 4**	*Examples of graphs associated to different cell-lines subjected to increasing values of radiation*	Pg. 5
**Supporting Information 5**	*Examining the sensitivity of the small-world-coefficient to the Waxman probability P*	Pg. 21

**Supporting Information 6**	*Examining the sensitivity of the small-world-coefficient to the Waxman probability P*	Pg. 21

Supporting Information 1. ***Image segmentation and analysis*.**

Fluorescence images (**Figure S1.1a**) of H4, H460, PC3 and T24 cells treated at 0, 2, 4, 6 Gy were segmented with Matlab® (2017b) to extract cell networks [1]. Images were converted to grayscale and low-pass filtered to remove constant power additive noise before being binarized with Otsu's method (**Figure S1.1b**) [2]. Morphological opening was performed to remove any small white noises in the image, and morphological closing to remove any small holes in the object. All connected components that had fewer than 8 pixels were removed and structures that were connected to the image border were suppressed. The images were segmented by a watershed transformation [3] and a distance transform [4] was used as segmentation function to split out the regions (**Figure S1.1c**). To avoid over-segmentation a minima imposition procedure [5] was implemented: tiny local minima were filtered out and the distance transform was modified so that no minima occurred at the filtered-out locations. With watershed segmentation, single cells were identified.

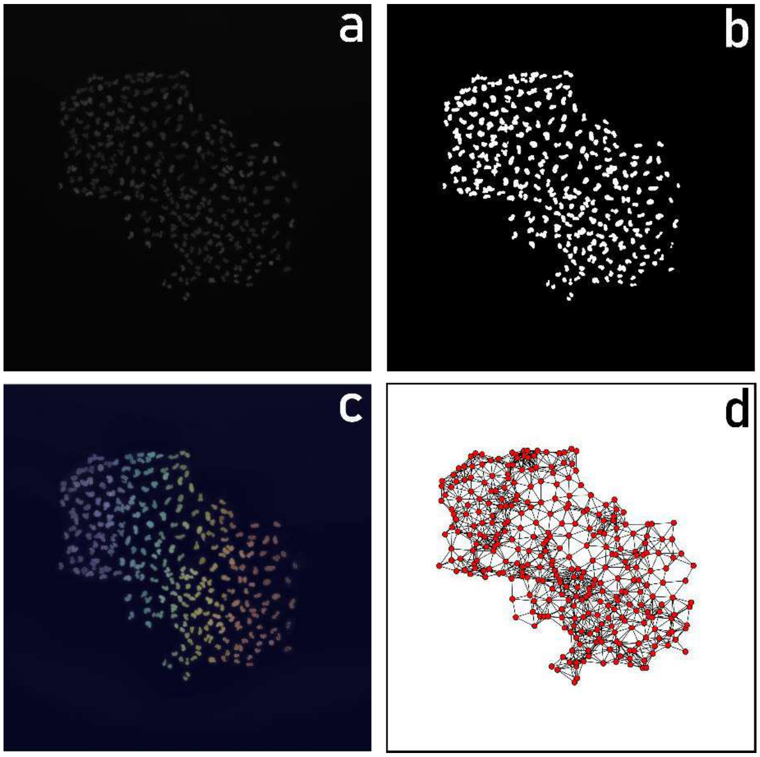

Figure S1.1. Image processing workflow and topological analysis. a) Fluorescence, b) binarized and c) watershed images; d) Connections between the cells with probability $p = 0.95$.

Supporting Information 2. ***Generating networks using the Waxman algorithm.***

In order to evaluate cell connectivity properties, we extracted network parameters such as the clustering coefficient (*cc*), the characteristic path length (*cpl*), and the small-world-ness (SW), as described in [1,6]. Briefly, the connections between the nodes of the network (**Figure S1.1d**) were established through the Waxman model [7], whereby the probability of being a link between two nodes u and v exponentially decreases with the Euclidean distance d between them:

$$p(u, v) = \alpha e^{-d(u,v)/\beta L} \qquad \text{(Eq.1)}$$

Where L is the largest possible Euclidean distance between two nodes of the grid and α and β are the Waxman model parameters that were set to 1 and 0.025, respectively. We decided whether a pair of nodes was connected using the following formula:

$$\alpha e^{-d_{i,j}/\beta L} - R \geq 0 \qquad \text{(Eq.2)}$$

in which R is a constant that we have chosen being 0.1, 0.05, 0.02 so that the probability of being a connection was $p = 0.9, 0.95, 0.98$.

Supporting Information 3. ***Topological analysis of cell networks.***

The information about the connections among the nodes of the graph was stored in the *adjacency matrix* $A=a_{ij}$, where the indices *i* and *j* run through the number of nodes *n* in the graph. In the analysis, reciprocity between nodes was assumed, and thus if information could flow from *i* to *j*, it could reversely flow from *j* to *i* (undirected graph).

With these premises, we extracted the local clustering coefficient as

$$C_i = \frac{2E_i}{b_i(b_i - 1)} \qquad \text{(Eq.3)}$$

Where b_i is the number of neighbors of a generic node *i*, E_i is the number of existing connections between those, $b_i(b_i - 1)/2$ is the maximum number of connections, or combinations, that can exist among *k* nodes. A global value, *cc*, was derived upon averaging C_i over all the nodes that composed the graph. Then, the characteristic path length (*cpl*) was determined as the average number of steps along the shortest paths for all possible pairs of network nodes. Once obtained *cc* and *cpl*, such values were compared with those extracted from equivalent Erdos-Rényi (*E–R*) random graphs to determine the small-world coefficient: indeed, a network G with *n* nodes and *m* edges is considered small-world if it has a similar path length but greater clustering of nodes than an equivalent Erdos-Rényi (*E–R*) random graph with the same *m* and *n* [8].

Finally, the network degree *k* was determined as the average number of links per node in a graph.

Supporting Information 4. ***Examples of graphs associated to different cell-lines subjected to increasing values of radiation*.**

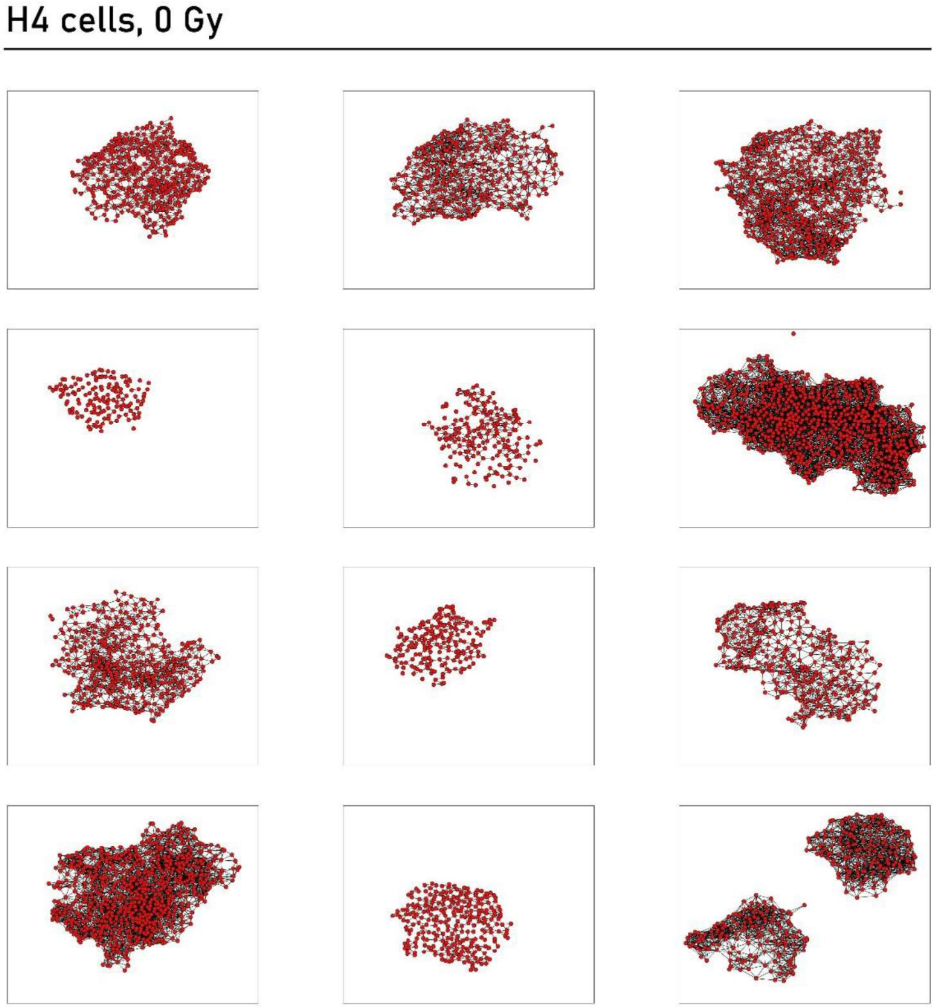

Figure S4.1. Examples of cancer-cell graphs associated to H4 cells subjected to 0 Gy. (Waxman parameters $\alpha = 1$, $\beta = 0.025$, $P = 0.95$).

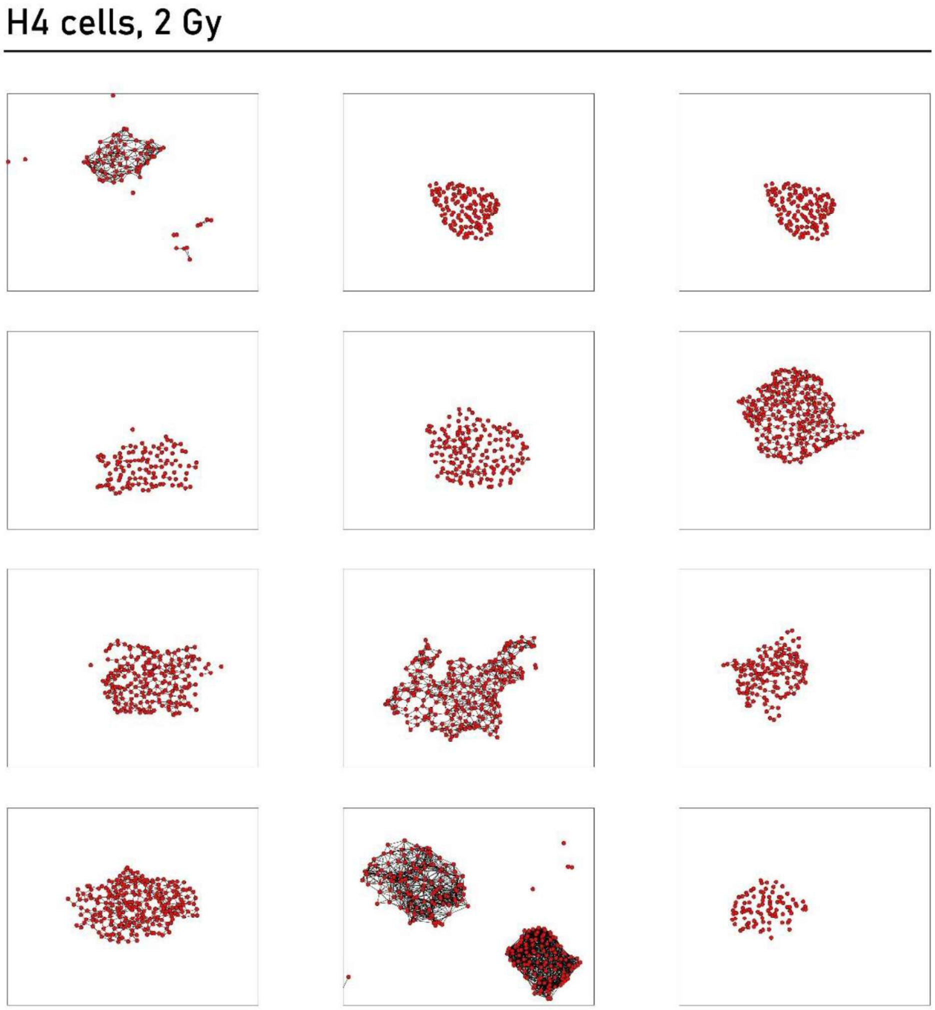

Figure S4.2. Examples of cancer-cell graphs associated to H4 cells subjected to 2 Gy. (Waxman parameters $\alpha = 1$, $\beta = 0.025$, $P = 0.95$).

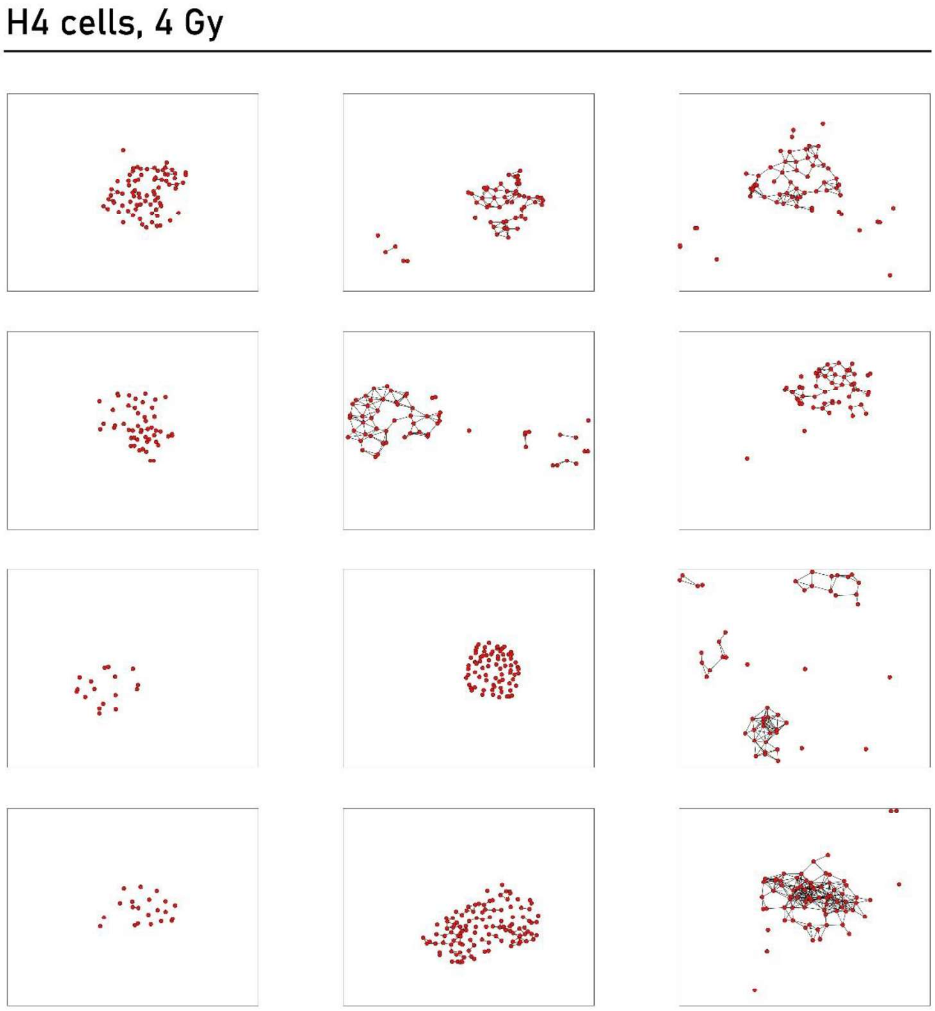

Figure S4.3. Examples of cancer-cell graphs associated to H4 cells subjected to 4 Gy. (Waxman parameters $\alpha = 1$, $\beta = 0.025$, $P = 0.95$).

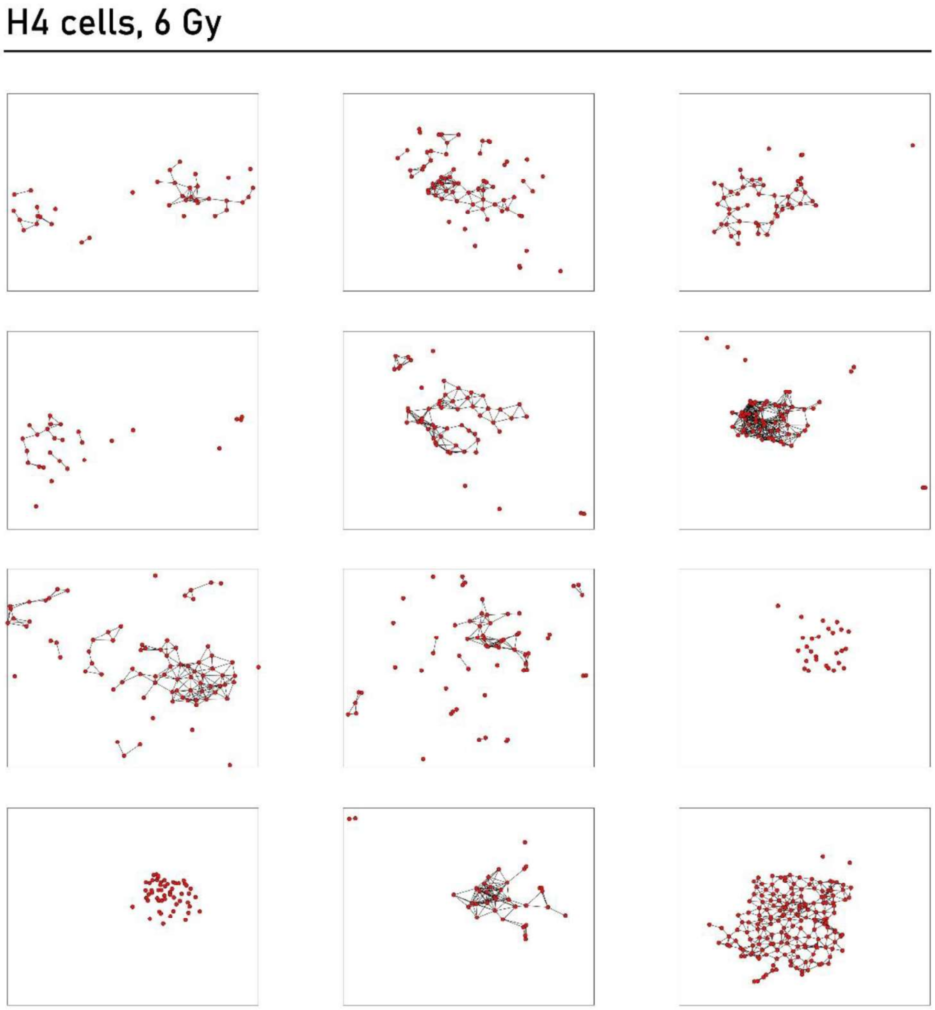

Figure S4.4. Examples of cancer-cell graphs associated to H4 cells subjected to 6 Gy. (Waxman parameters $\alpha = 1$, $\beta = 0.025$, $P = 0.95$).

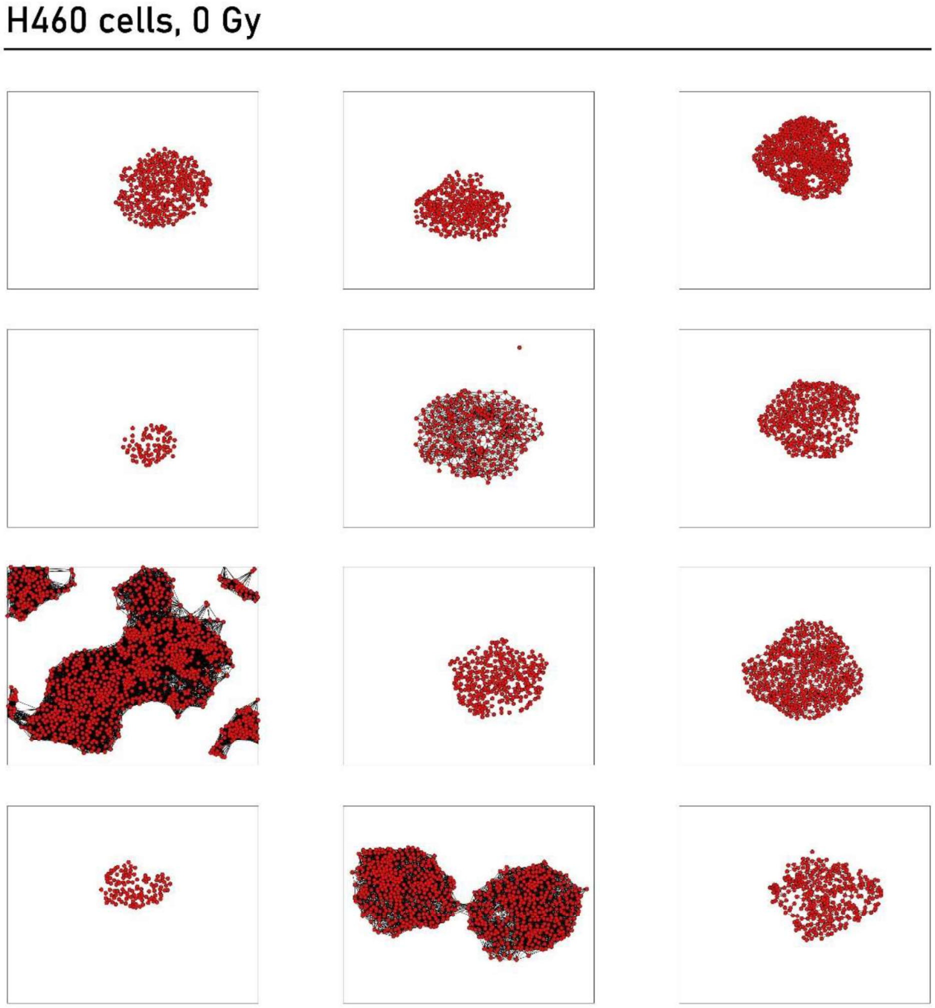

Figure S4.5. Examples of cancer-cell graphs associated to H460 cells subjected to 0 Gy. (Waxman parameters $\alpha = 1$, $\beta = 0.025$, $P = 0.95$).

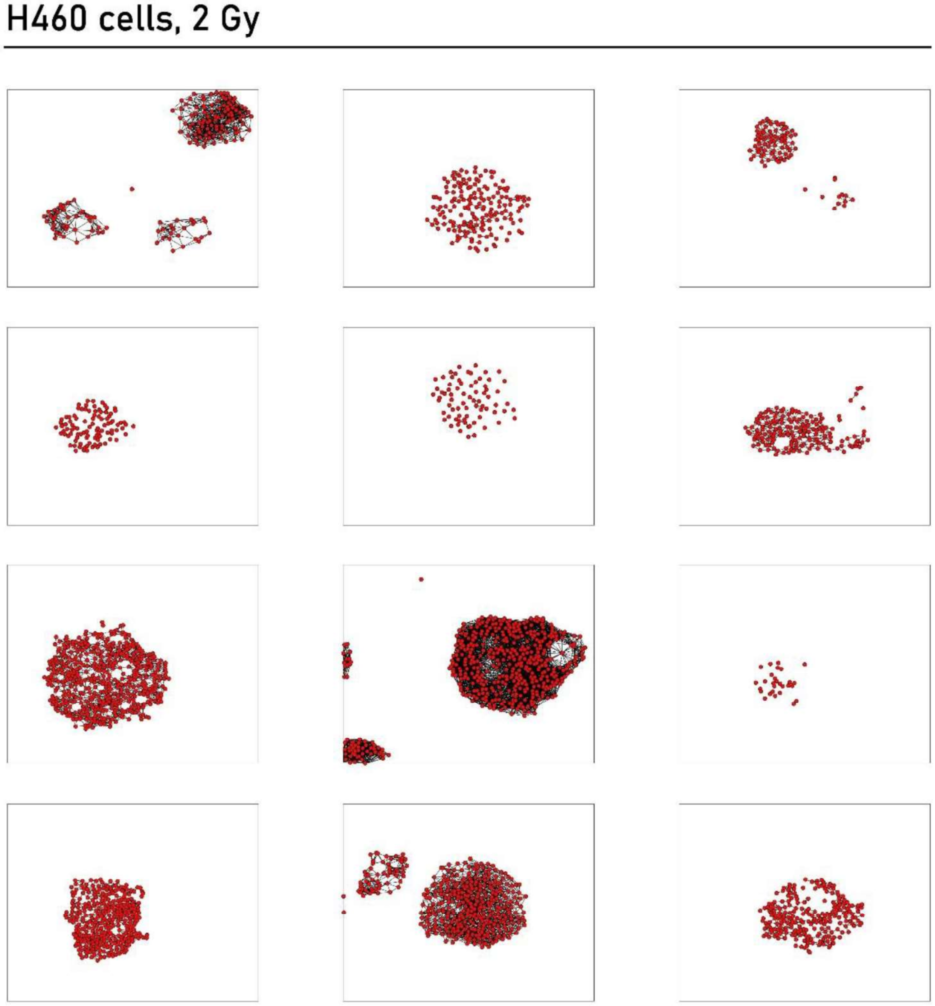

Figure S4.6. Examples of cancer-cell graphs associated to H460 cells subjected to 2 Gy. (Waxman parameters $\alpha = 1, \beta = 0.025, P = 0.95$).

H460 cells, 4 Gy

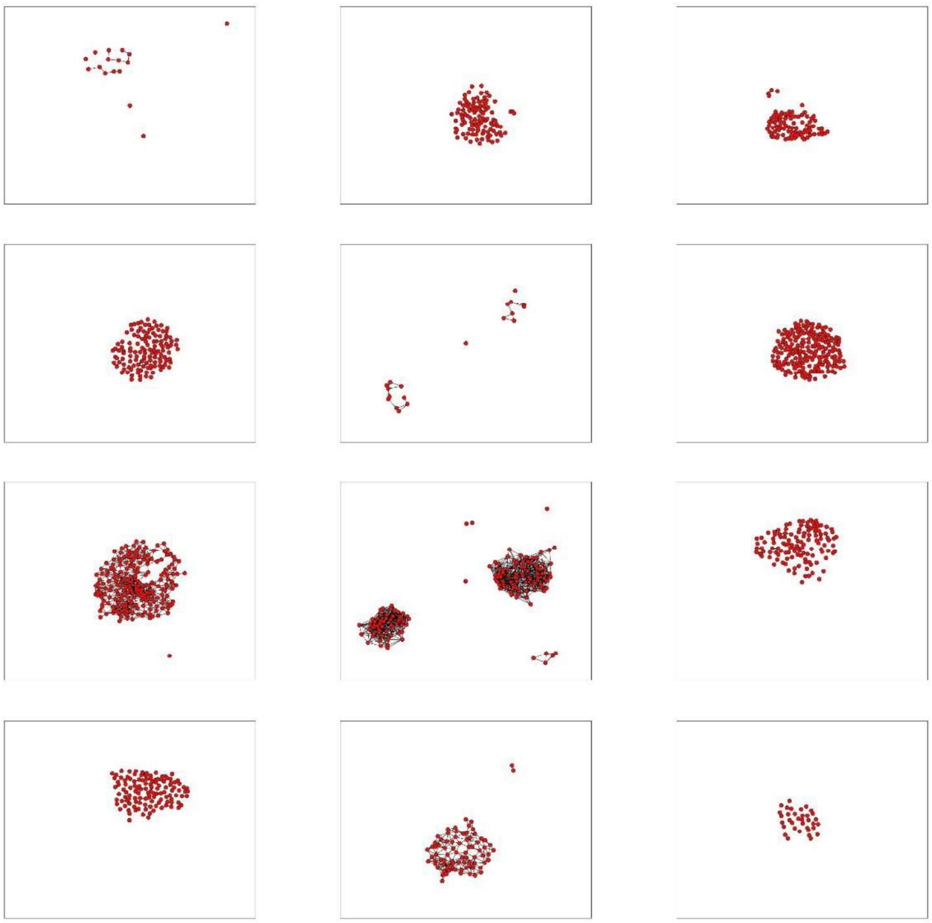

Figure S4.7. Examples of cancer-cell graphs associated to H460 cells subjected to 4 Gy. (Waxman parameters $\alpha = 1$, $\beta = 0.025$, $P = 0.95$).

H460 cells, 6 Gy

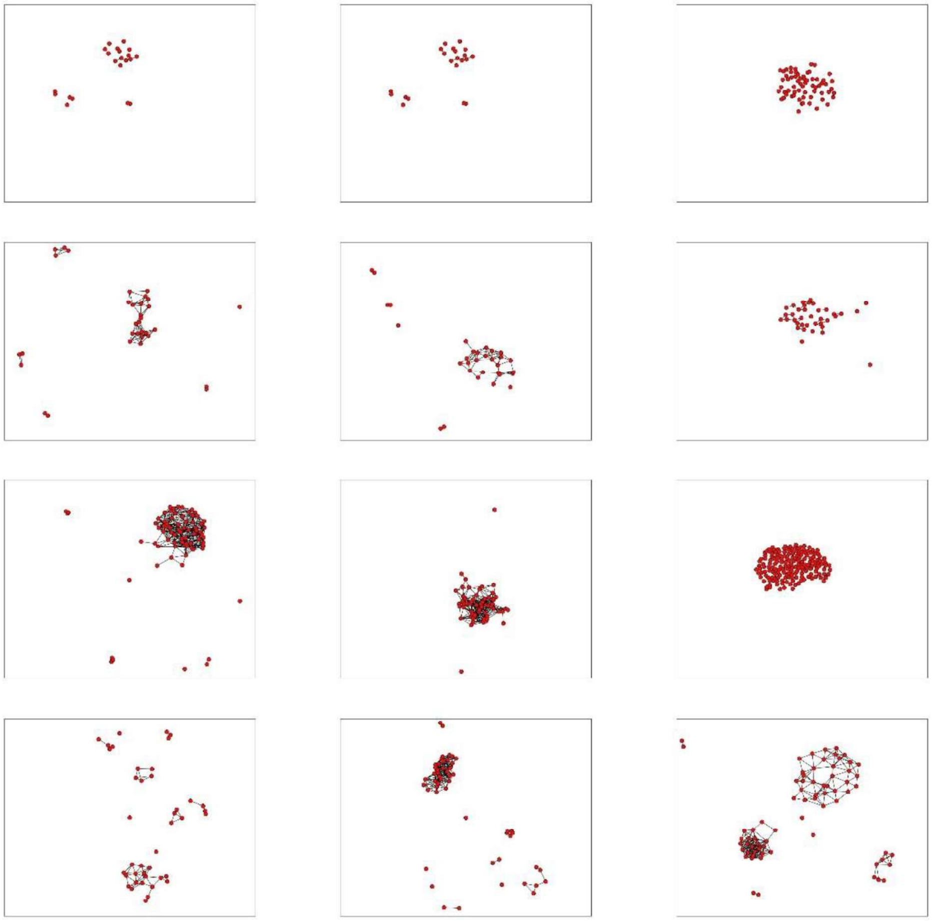

Figure S4.8. Examples of cancer-cell graphs associated to H460 cells subjected to 6 Gy. (Waxman parameters $\alpha = 1$, $\beta = 0.025$, $P = 0.95$).

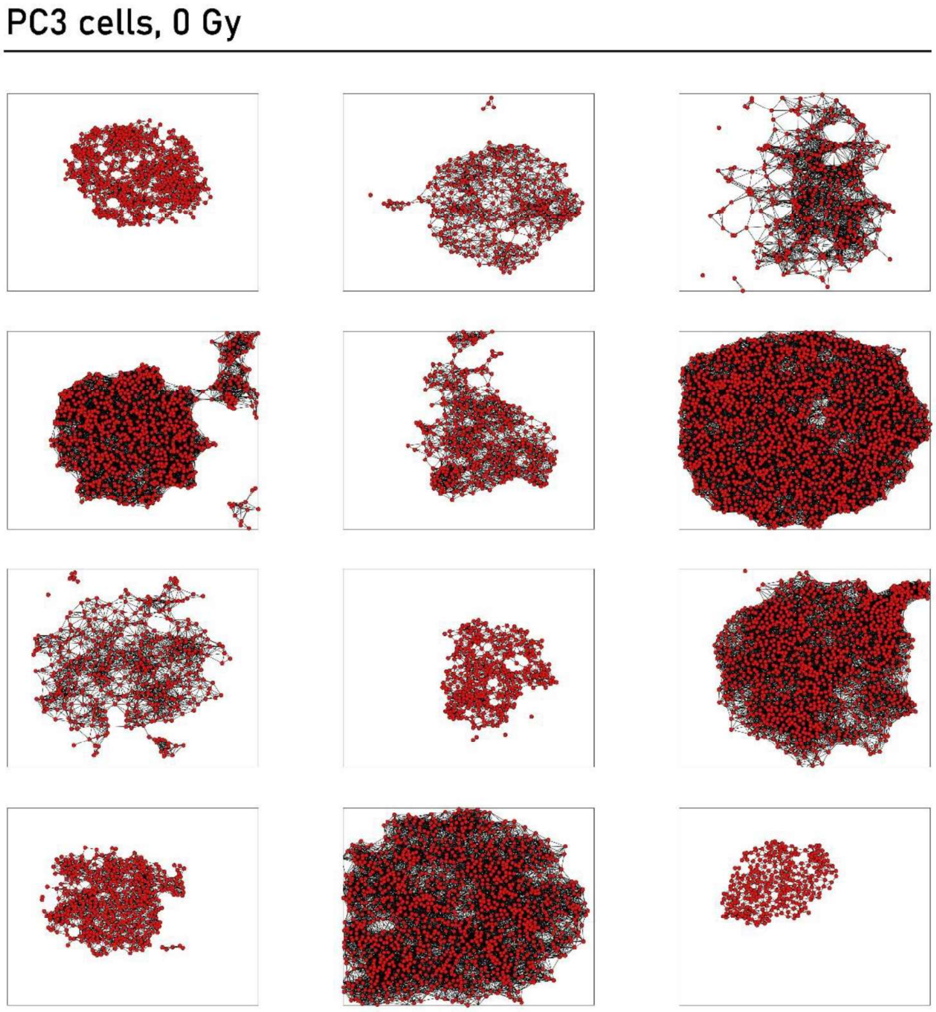

Figure S4.9. Examples of cancer-cell graphs associated to PC3 cells subjected to 0 Gy. (Waxman parameters $\alpha = 1$, $\beta = 0.025$, $P = 0.95$).

PC3 cells, 2 Gy

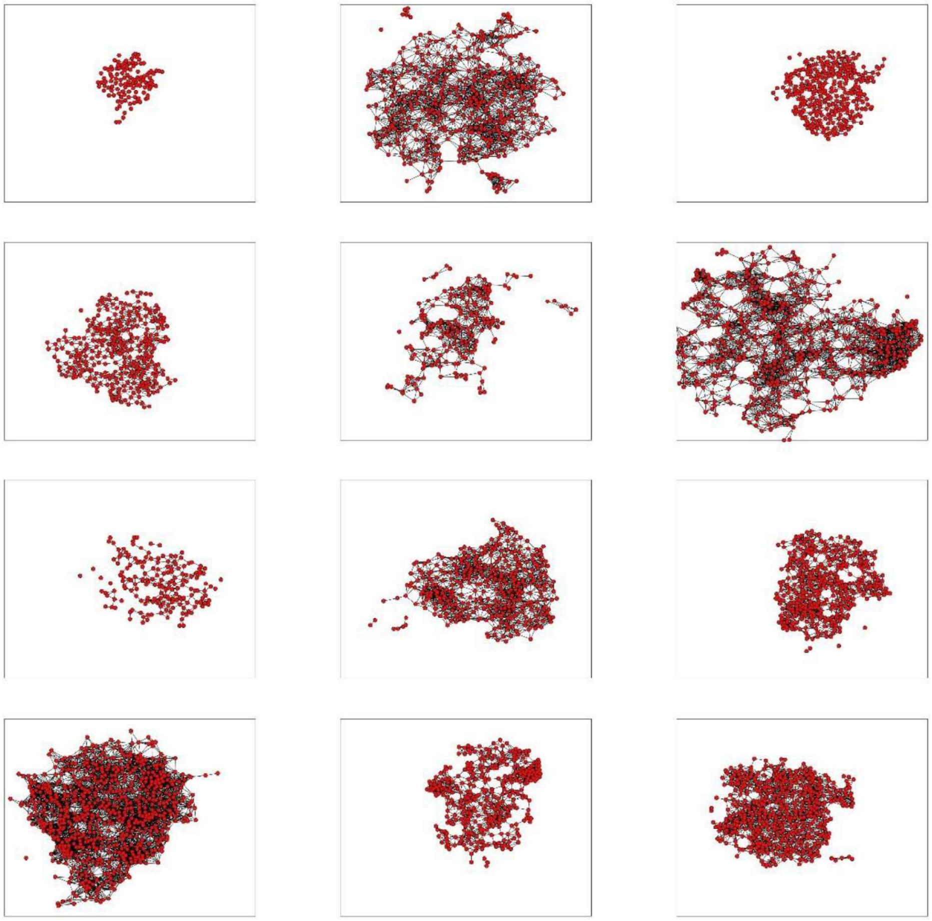

Figure S4.10. Examples of cancer-cell graphs associated to PC3 cells subjected to 2 Gy. (Waxman parameters $\alpha = 1$, $\beta = 0.025$, $P = 0.95$).

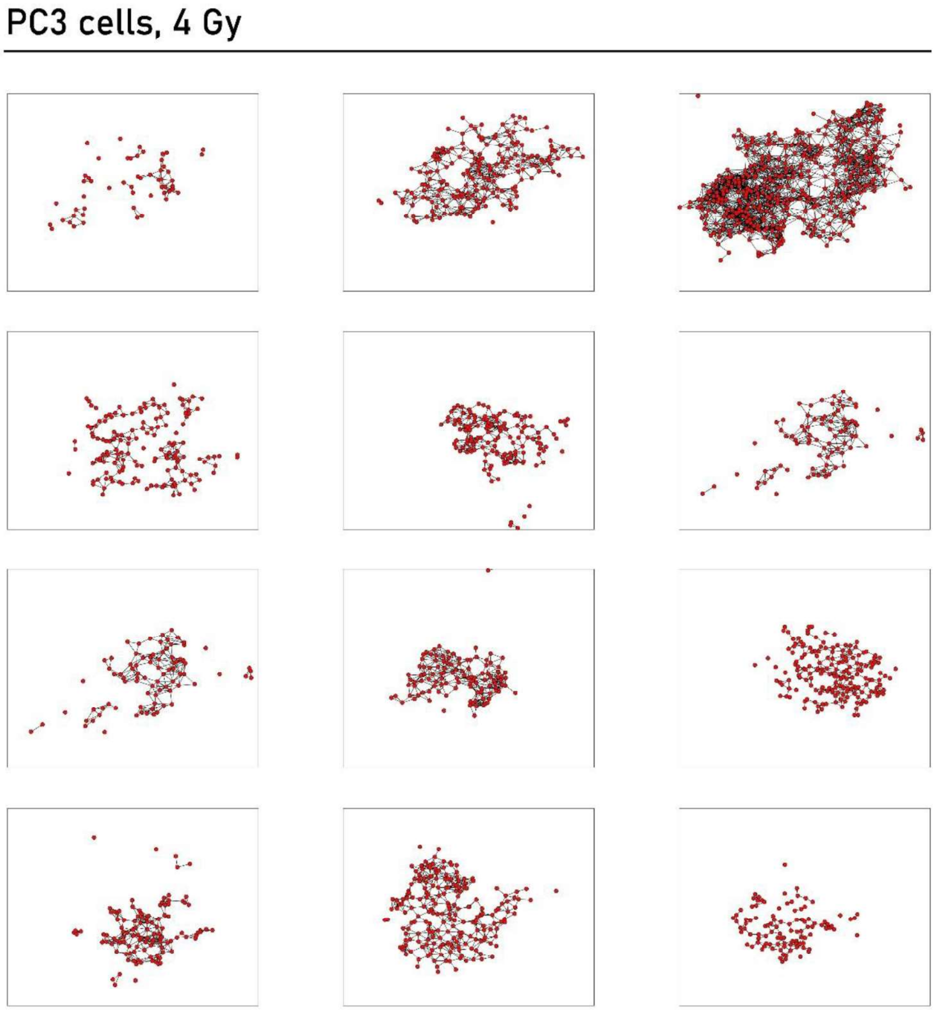

Figure S4.11. Examples of cancer-cell graphs associated to PC3 cells subjected to 4 Gy. (Waxman parameters $\alpha = 1$, $\beta = 0.025$, $P = 0.95$).

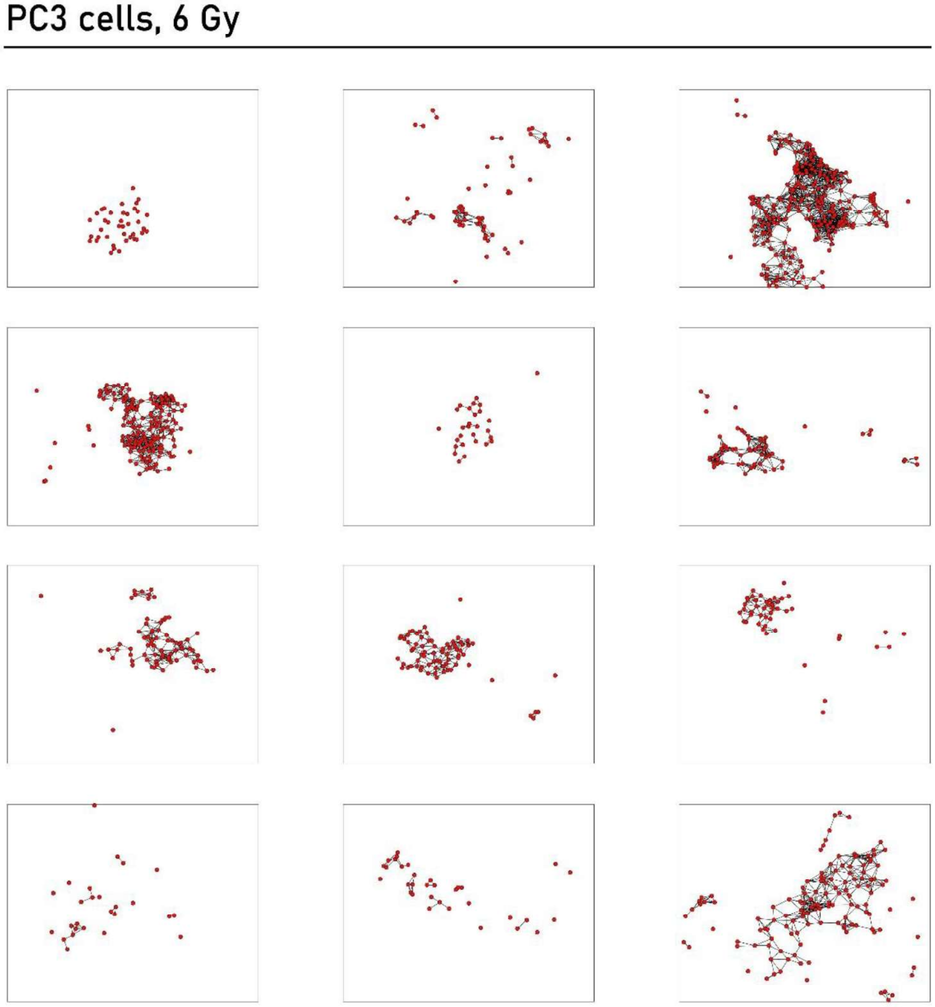

Figure S4.12. Examples of cancer-cell graphs associated to PC3 cells subjected to 6 Gy. (Waxman parameters $\alpha = 1, \beta = 0.025, P = 0.95$).

T24 cells, 0 Gy

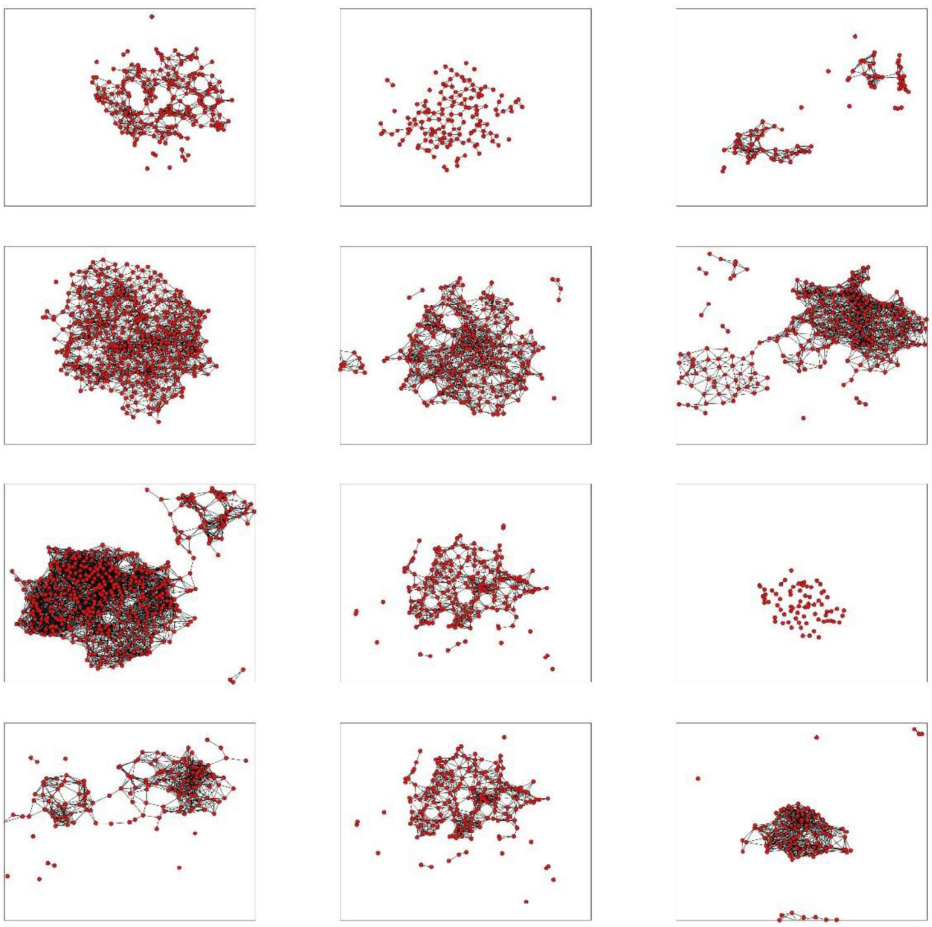

Figure S4.13. Examples of cancer-cell graphs associated to T24 cells subjected to 0 Gy. (Waxman parameters $\alpha = 1$, $\beta = 0.025$, $P = 0.95$).

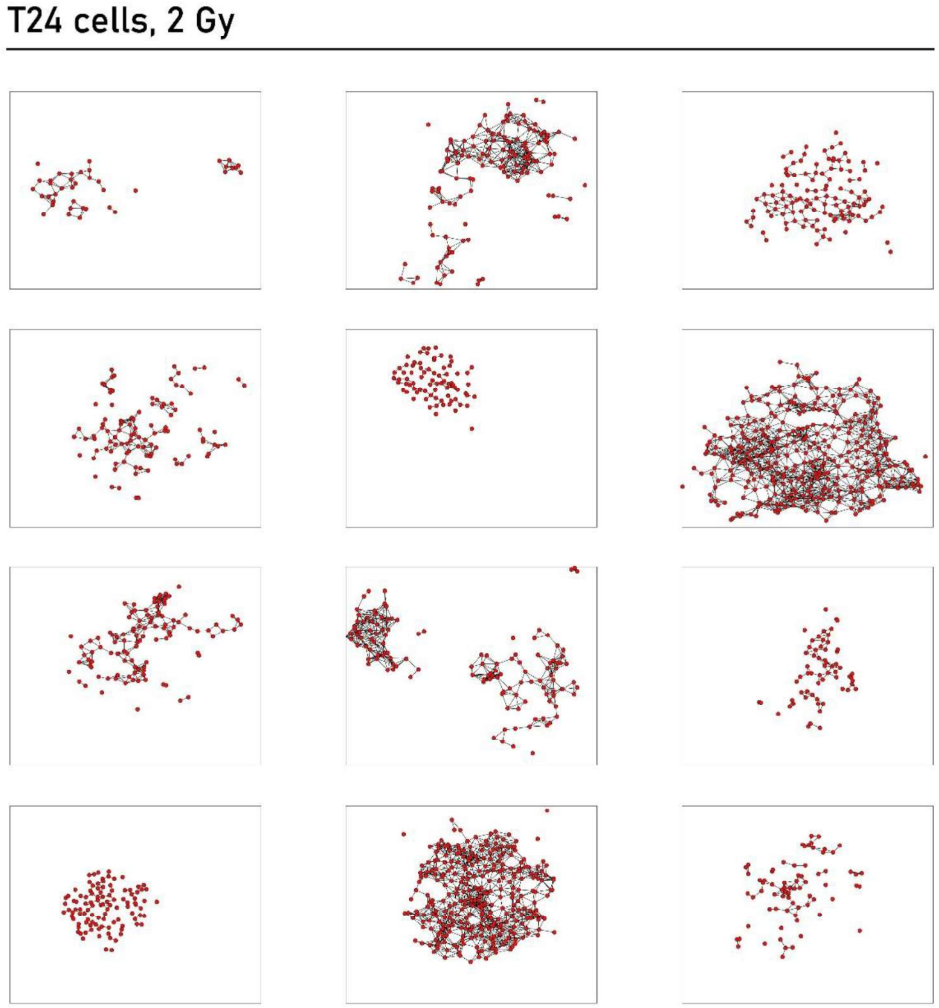

Figure S4.14. Examples of cancer-cell graphs associated to T24 cells subjected to 2 Gy. (Waxman parameters $\alpha = 1$, $\beta = 0.025$, $P = 0.95$).

T24 cells, 4 Gy

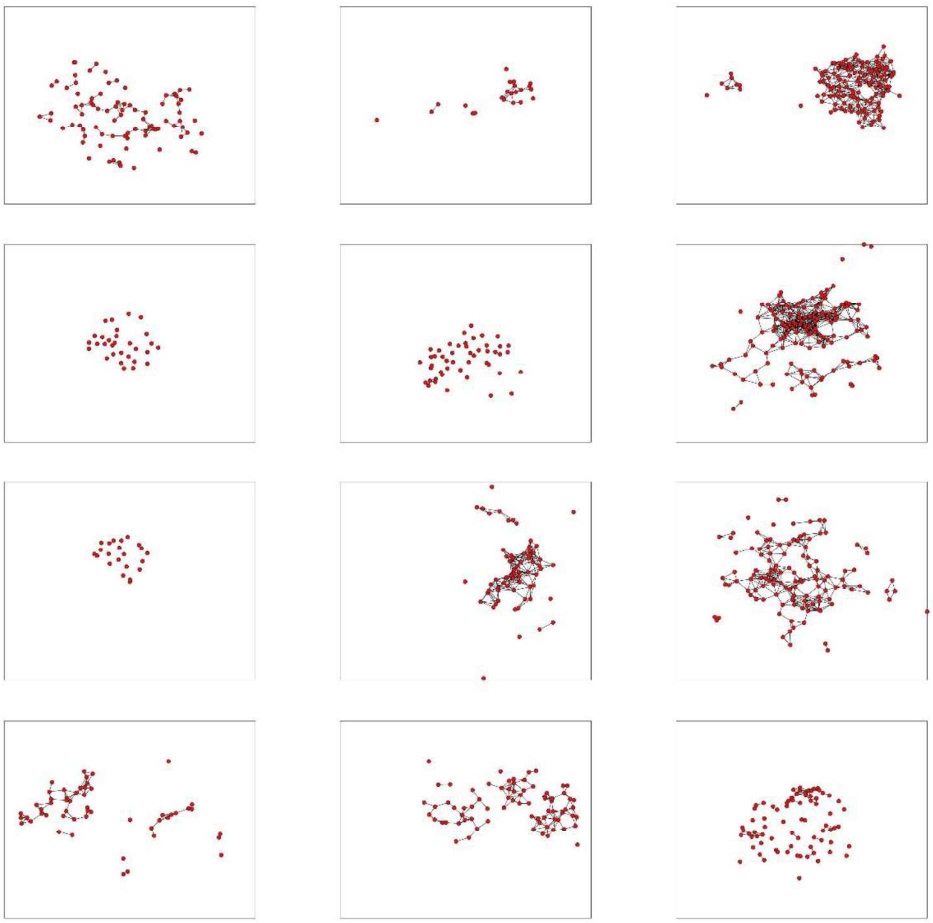

Figure S4.15. Examples of cancer-cell graphs associated to T24 cells subjected to 4 Gy. (Waxman parameters $\alpha = 1$, $\beta = 0.025$, $P = 0.95$).

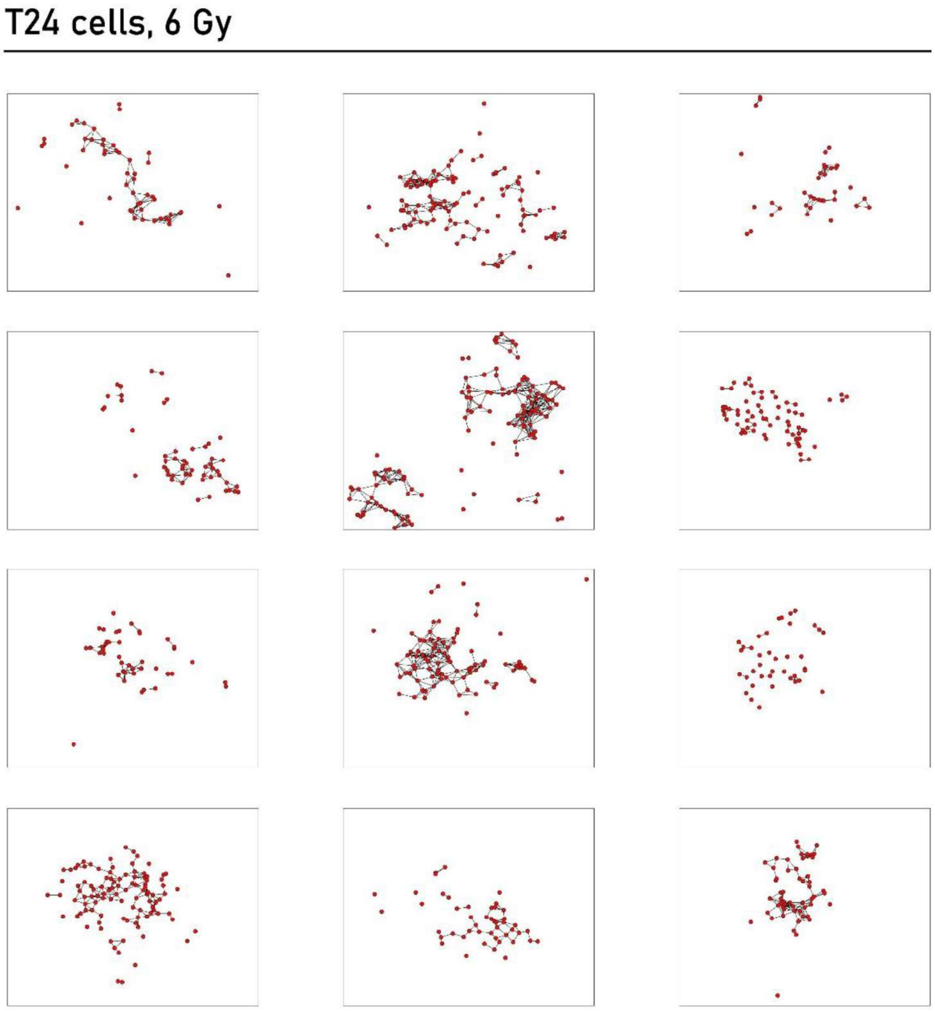

Figure S4.16. Examples of cancer-cell graphs associated to T24 cells subjected to 6 Gy. (Waxman parameters $\alpha = 1$, $\beta = 0.025$, $P = 0.95$).

Supporting Information 5. ***Statistical analysis*.**

We have calculated for each statistical analysis performed in this study the values of sample size, P-value, t-stat, and degree of freedom, recapitulated in the following as:

Cell line/dose	0 Gy	2 Gy	4 Gy	6 Gy
H4	135	119	97	118
H460	106	111	114	97
PC3	132	125	102	114
T24	74	105	103	94

Table S5.1. ***Sample size.***

Dose/Dose	0 Gy	2 Gy	4 Gy	6 Gy
0 Gy	-	-	0.0032<br>-5.2362<br>99	0.0063<br>-6.31691<br>99
2 Gy	-	-	-	-
4 Gy	0.0032<br>-5.2362<br>99	-	-	-
6 Gy	0.0063, -6.31691<br>99	-	-	-

Table S5.2. ***H4 cells*** – *Student's T-test comparison between sample groups*: P-value, t-stat and degree of freedom resulting from the comparison of samples subjected to different values of dose.

Dose/Dose	0 Gy	2 Gy	4 Gy	6 Gy
0 Gy	-	-	0.035<br>-1.28122<br>99	0.004<br>-4.52024<br>99
2 Gy	-	-	-	-
4 Gy	0.035<br>-1.28122<br>99	-	-	-
6 Gy	0.004<br>-4.52024<br>99	-	-	-

Table S5.3. ***H460 cells*** – *Student's T-test comparison between sample groups*: P-value, t-stat and degree of freedom resulting from the comparison of samples subjected to different values of dose.

Dose/Dose	0 Gy	2 Gy	4 Gy	6 Gy
0 Gy	-	-	-	0.00910<br>2.03136<br>99
2 Gy	-	-	-	-
4 Gy	-	-	-	-
6 Gy	0.00910<br>2.03136<br>99	-	-	-

Table S5.4. ***PC3 cells*** *– Student's T-test comparison between sample groups*: P-value, t-stat and degree of freedom resulting from the comparison of samples subjected to different values of dose.

Dose/Dose	0 Gy	2 Gy	4 Gy	6 Gy
0 Gy	-	0.01900<br>-2.36477<br>99	0.00066<br>-3.51402<br>99	0.00050<br>-3.59899<br>99
2 Gy	0.01900<br>-2.36477<br>99	-	-	-
4 Gy	0.00066<br>-3.51402<br>99	-	-	-
6 Gy	0.00050<br>-3.59899<br>99	-	-	-

Table S5.5. ***T24 cells*** *– Student's T-test comparison between sample groups*: P-value, t-stat and degree of freedom resulting from the comparison of samples subjected to different values of dose.

Supporting Information 6. ***Examining the sensitivity of the small-world-coefficient to the Waxman probability P.***

The mean sw-coefficient of cancer-cell graphs as a function of the exposing dose, determined for different cancer-cell lines (H4, H460, PC3, T24) and for different values of the model parameter P in the network-Waxman generator model ($P = 0.9$, $P = 0.95$, $P = 0.98$) (**Figure S6.1**). (The remaining parameters in the Waxman model have been set as $\alpha = 1$, $\beta = 0.025$).

The small-world coefficient of cancer cell graphs varies as a function of the cut-off probability P – for different values of the radiation dose (**Figure S6.2**). Diagrams illustrate that the trend of sw depends on cell type and the dose. For smaller values of the dose comprised between 0 and 2 Gy, the sw coefficient increases moderately with P. In contrast, for larger values of the dose (4, 6 Gy) the sw coefficient decreases moderately with P, with the exception, notably, of the H460 cells – for which sw always increases with the probability P regardless of the dose.

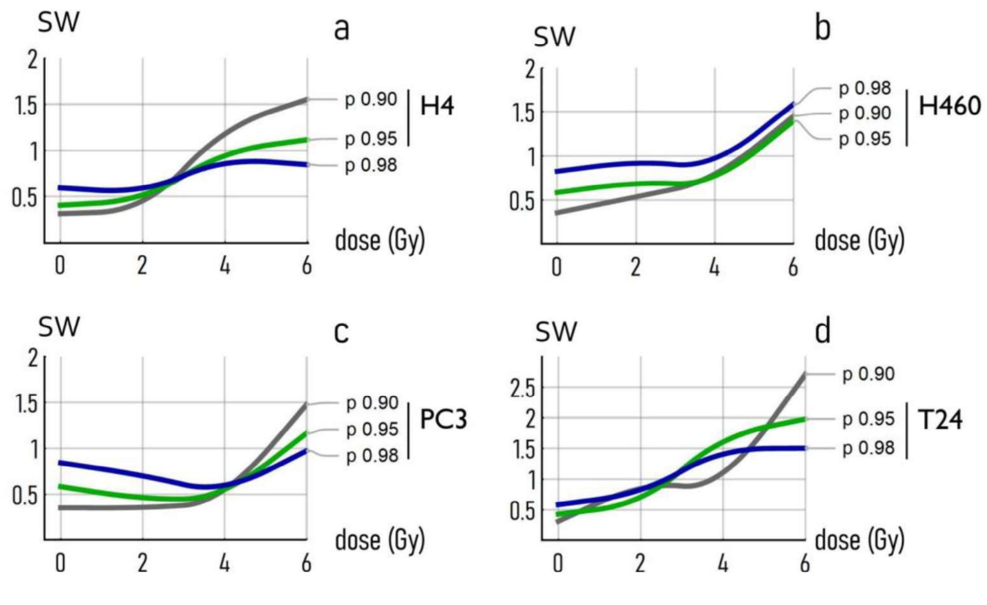

Figure S6.1

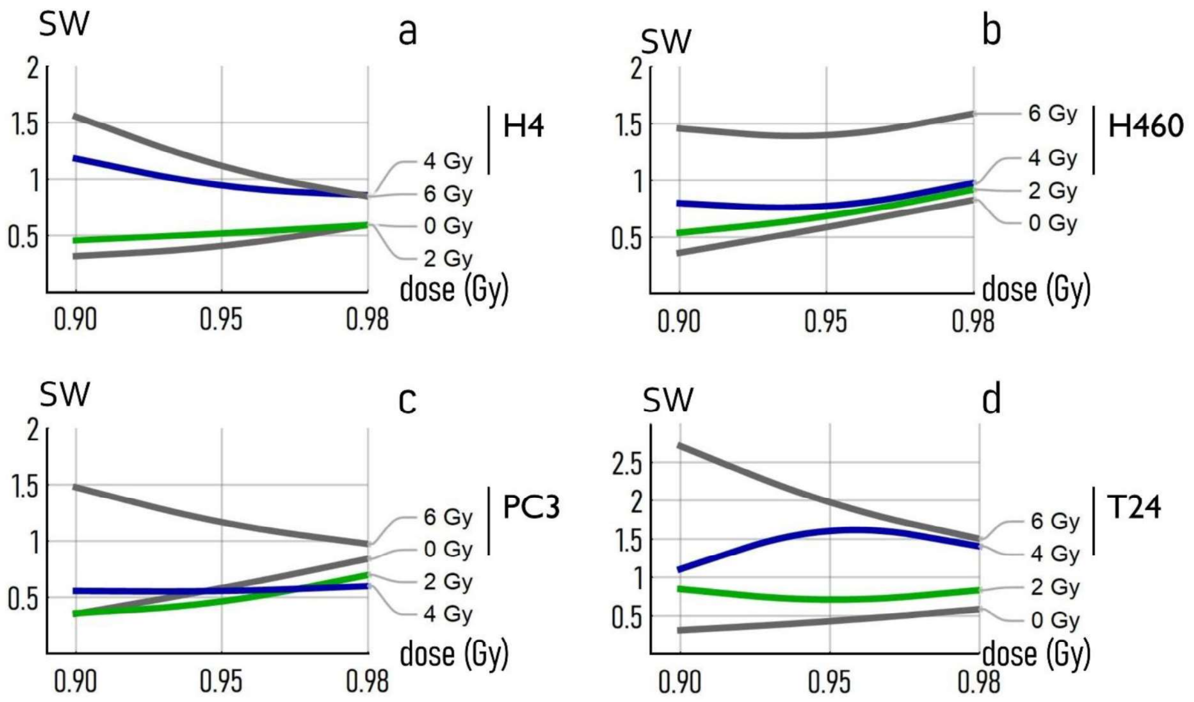
SW
a
H4
4 Gy
6 Gy
0 Gy
2 Gy
dose (Gy)
0.90
0.95
0.98
SW
b
H460
6 Gy
4 Gy
2 Gy
0 Gy
dose (Gy)
SW
c
PC3
6 Gy
0 Gy
2 Gy
4 Gy
dose (Gy)
SW
d
T24
6 Gy
4 Gy
2 Gy
0 Gy
dose (Gy)

Figure S6.2

References

1. Onesto V, Villani M, Narducci R, et al. Cortical-like mini-columns of neuronal cells on zinc oxide nanowire surfaces. Scientific Reports. 9:4021 2019.
2. Otsu N. A Threshold Selection Method from Gray-Level Histograms. IEEE Transactions on Systems, Man, and Cybernetics. 9:62-66, 1979.
3. Meyer F. Topographic distance and watershed lines. Signal Processing. 38:113-125, 1994.
4. Maurer C, Qi R, Raghavan V. A Linear Time Algorithm for Computing Exact Euclidean Distance Transforms of Binary Images in Arbitrary Dimensions. IEEE Transactions on Pattern Analysis and Machine Intelligence. 25:265-270, 2003.
5. Vincent L. Morphological Grayscale Reconstruction in Image Analysis: Applications and Efficient Algorithms. IEEE Transactions on Image Processing. 2:176-201, 1993.
6. Onesto V, Cancedda L, Coluccio M, et al. Nano-topography Enhances Communication in Neural Cells Networks. Scientific Reports. 7:1-13, 2017.
7. Waxman B. Routing of multipoint connections. IEEE Journal on Selected Areas in Communications. 6:1617–1622, 1988.
8. Humphries MD, Gurney K. Network ‘Small-World-Ness’: A Quantitative Method for Determining Canonical Network Equivalence. PLoS ONE. 3:e0002051, 2008.
